# Molecular Targets and Signaling Pathways of microRNA-122 in Hepatocellular Carcinoma

**DOI:** 10.3390/pharmaceutics14071380

**Published:** 2022-06-29

**Authors:** Kwang-Hoon Chun

**Affiliations:** Gachon Institute of Pharmaceutical Sciences, College of Pharmacy, Gachon University, Incheon 21936, Korea; khchun@gachon.ac.kr; Tel.: +82-32-820-4951

**Keywords:** miR-122, hepatocellular carcinoma, molecular targets, signaling pathways, metastasis

## Abstract

Hepatocellular carcinoma (HCC) is one of the leading global causes of cancer mortality. MicroRNAs (miRNAs) are small interfering RNAs that alleviate the levels of protein expression by suppressing translation, inducing mRNA cleavage, and promoting mRNA degradation. miR-122 is the most abundant miRNA in the liver and is responsible for several liver-specific functions, including metabolism, cellular growth and differentiation, and hepatitis virus replication. Recent studies have shown that aberrant regulation of miR-122 is a key factor contributing to the development of HCC. In this review, the signaling pathways and the molecular targets of miR-122 involved in the progression of HCC have been summarized, and the importance of miR-122 in therapy has been discussed.

## 1. Introduction

Primary liver cancer (PLC) is a high-risk malignant tumor with approximately 906,000 new cases diagnosed in 2020, accounting for 4.7% of all cancer cases [1]. Hepatocellular carcinoma (HCC) is the most common type of PLC, accounting for approximately 75–85% of PLC cases [2]. HCC is the third leading cause of cancer mortality worldwide, leading to approximately 830,000 deaths in 2020 [1]. Unfortunately, most of the patients are diagnosed at a later or more advanced stage, wherein effective therapy is still unavailable. Thus, the molecular mechanisms involved in the pathogenesis of HCC must be elucidated. In most of the cases, HCC usually develops from liver fibrosis and cirrhosis caused by risk factors such as chronic viral infection, lipid accumulation, alcohol consumption, and toxins [3]. The major types of etiological factors include the hepatitis viruses B (HBV) and C (HCV), which contribute to 16% and 48% of HCC cases in the United States, respectively [4].

MicroRNAs are endogenously expressed noncoding RNAs that regulate the translational efficiency or downregulation of target mRNAs. Since the discovery of the let-7 miRNA in *C. elegans* [5], more than 38,000 entries have been enrolled in miRbase Release 22.1 as of March 2022 [6]. An miRNA functions via the RNAi pathway, guiding the RNA-induced silencing complex to mRNA by Watson–Crick base pairing [7], which results in transcriptional or translational repression of the target mRNA. In most animals, sequence complementarity between a miRNA and its target mRNA is not perfect. Most of the binding sites contain mismatches between the complementary strands, which play an important role in target gene suppression [8,9]. Imperfect complementarity allows an miRNA to bind to a variable number of sites, thus endowing a great diversity of target mRNAs [10].

miR-122 is one of the first examples of a tissue-specific miRNA. The expression of miR-122 is specific to the liver tissues of vertebrate lineages [11] and is abundantly present, accounting for approximately 72% of all cloned miRNAs from mouse livers [12]. The miRbase suggests 5′-UGGAGUGUGACAAUGGUGUUUG-3′ as a consensus sequence of miR-122-5p in both humans and mice [6].

Since the discovery of miR-122 in mammals in 1989 [13], its diverse biological roles have been identified. Interestingly, it was observed that mouse models with germline knockouts (KOs) or a liver-specific KO (LKO) of miR-122 were still viable and fertile, indicating that miR-122 could not be an indispensable regulator of liver function despite its abundant hepatic expression. However, aged mice with genetic defects developed fibrosis, steatohepatitis, and HCC, expressing abnormally genes involved in cellular growth, cell death, epithelial–mesenchymal transition, and cancer, indicating the antitumor ability of miR-122 [14,15].

Additionally, several knockdown studies using antisense oligonucleotides or antagomirs showed a reduction in the total cholesterol, low-density lipoprotein, high-density lipoprotein, and serum triacylglycerol levels, implicating the potential of anti-miR-122 therapeutics for the treatment of metabolic disorders [16,17,18]. Unfortunately, antitumor activity has not been evaluated in these knockdown studies; these studies were designed based on normal or obese mouse models, but not cancer models, and the animals were also treated with miR-122 inhibitors over relatively short periods [19]. 

Meanwhile, the target identification studies performed by Boutz D.R. et al. coincided well with the results obtained from loss-of-function studies. Herein, miR-122 targets were screened by combining the luciferase reporter system and shotgun proteomics, which resulted in the identification of 260 downregulated proteins, 113 genes of which contained predicted miR-122 target sites. Importantly, an enrichment assay performed for these target proteins revealed that miR-122 is related to various functions, such as cell proliferation, differentiation, and cell death. Additionally, parts of these targets are associated with liver metabolism and diseases, including HCC [20].

**Figure 1:**
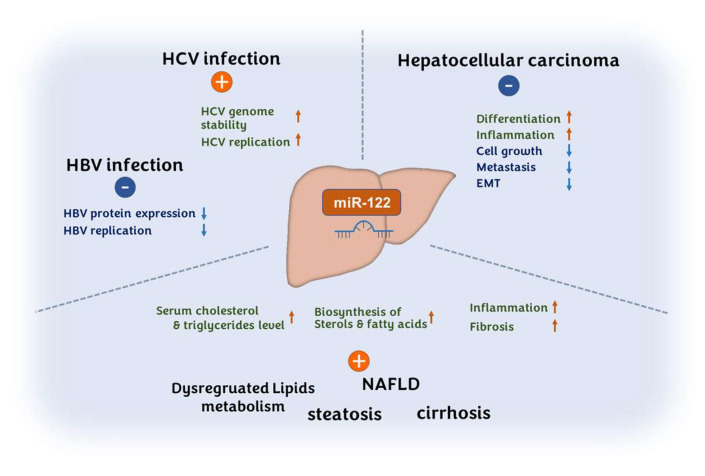
miR-122 is involved in the development of viral infections, hepatocellular carcinoma, and various metabolic conditions. miR-122 negatively regulates the pathogenic progress of hepatocellular carcinoma; promotes hepatitis C viral proliferation while suppressing hepatitis B viral proliferation; increases the biosynthesis of sterols and fatty acids; and accelerates the progression of inflammation and fibrosis, leading to lipid-related metabolic disorders.

The activation of miR-122 is associated with several liver diseases, including HCV infections [21,22,23], HBV infections [24], steatosis, and cirrhosis [23,25,26,27,28,29]. In HCV, miR-122 plays an important role as a host factor for viral replication by increasing HCV translation and genomic RNA stability [11,30,31,32]. For this reason, a miR-122 inhibitor, miravirsen, was the first miRNA-based therapeutic that entered the clinic [33]. The miR-122-related diverse disorders and the roles of miR-122 are illustrated in Figure 1.

## 2. miR-122 in HCC 

Recent accumulating evidence suggests the involvement of miR-122 in the tumorigenesis, invasiveness, and metastasis of hepatocellular carcinomas (HCCs). Several previous studies have reported that the miR-122 levels were downregulated in the serum of HCC patients [34,35,36] as well as in liver tumor tissues [37,38,39,40,41,42,43,44,45,46,47,48]. The expression profile data of miR-122 in HCC patients (1330 HCC vs. 735 control) obtained from The Cancer Genome Atlas (TCGA) and the Gene Expression Omnibus revealed the downregulation of miR-122 in HCC tissues [49]. Moreover, the serum and plasma levels of miR-122 were downregulated in HBV-infected HCC patients [50,51] (reviewed in [52,53]). It was also found that miR-122 was downregulated in the liver of chronic hepatitis B patients [54,55], HBV-infected cells, and HBV transgenic mice [56]. Specifically, a recent meta-analysis of serum miR-122 levels in HCC analyzed 13 studies with 920 HCC patients (vs. 1217 controls), and showed that miR-122 can discriminate the HCC patients from the control patients with a high sensitivity (0.76, 95% CI: 0.69–0.81) and specificity (0.75, 95% CI: 0.67–0.82) [57]. Consistently, another meta-analysis including 354 HCV-infected HCC patients (vs. 420 control) also showed that the levels of miR-122 in the serum (five studies) and plasma samples (one study) were downregulated [58].

Although reported only minorly, several studies have reported contradictory results showing no significant difference or even the upregulation of serum [59,60,61,62] and tissue [63] miR-122 levels in HCC patients. One possible explanation, albeit not yet clearly defined, is that miR-122 expression seems to be differentially regulated by the heterogeneity of samples under different environmental conditions, such as stage-dependent changes in the miR-122 expression levels during HBV infection [60]. Coincidently, heterogenic characters of miR-122 expression have been reported to depend on the experimental contexts. For instance, an extreme variation in the expression levels of miR-122 was reported among HCC-derived cell lines, showing more than a 1000-fold difference. PLC/PRF/5, Huh-1, and Hep40 cell lines showed very high expression of miR-122, whereas Hep3B and HepG2 expressed very low levels of miR-122, and no expression was detected in SNU387 and SNU398 [64]. Furthermore, the miR-122 expression levels were correlated with the HCC metastatic stage. It was observed that the miR-122 level in tumor tissues was remarkably lower in the T3 stage with intrahepatic metastasis compared with those in the adjacent normal tissues and the T1 stage [65]. 

Despite these minor discrepancies, the anticancer activity of miR-122 in HCC has been further supported by the findings that overexpressed miR-122 reduces cell migration and invasion in HCC-derived cells and local invasion in vivo [43,65]. These studies indicate the validity of the administration of miR-122 in HCC treatment. This has been supported by numerous studies showing that miR-122 administration can suppress HCC tumorigenicity and metastasis in vivo [43,66,67] and enhance the anticancer activity of clinically prescribed drugs as combinatorial therapy [43,68,69,70,71,72]. Additionally, lower levels of miR-122 in HCC have been associated with a poor prognosis [64,73].

Given the importance of miR-122 in HCC progression, more extensive research on the mechanisms involved with miR-122-related HCC would improve the understanding of the etiological mechanisms in HCC development and the discovery of new therapeutic approaches. This article summarizes the molecular targets of miR-122 that are involved in HCC progression and discusses the related signaling pathways to further enhance the understanding of miR-122 in HCC progression.

## 3. Endogenous Targets and Signaling Pathways of miR-122 in HCC

Here, the known targets of miR-122 that are involved in HCC progression are described, and their roles and signaling pathways are systemically summarized. As previously noted, there has been convincing evidence depicting the role of miR-122 as a tumor suppressor gene. Its downregulation could promote tumor formation, whereas the addition of miR-122 into HCC cells could reverse the tumorigenic properties of cancer cells. This antitumor activity of miR-122 can be analyzed by a network of miR-122 target genes involved in diverse functions such as cell proliferation, apoptosis, glycolytic and lipid metabolism, and mutagenesis.

### 3.1. Cell Growth, Survival, and Antiapoptotic Signaling 

Many studies have shown that miR-122 exhibits its antitumor activity by inhibiting cell growth but inducing cell cycle arrest and apoptosis in cancer cells. Numerous targets of miR-122 have been reported to be key regulators in the cell cycle and cell death signaling pathways. It has been suggested that miR-122 could be used as an effective combinatorial agent with radiotherapeutics [74] and chemotherapeutics [66,68] by inducing cell cycle arrest or apoptosis. Additionally, the suppressing activity of miR-122 against the levels of the multidrug resistance (MDR)-related gene MDR-1, glutathione S-transferase π, and MDR-associated proteins could enhance the sensitivity of HCC cells to chemotherapeutic agents [69].

#### 3.1.1. Insulin-like Growth Factor-1 Receptor (IGF-1R)

The binding of insulin-like growth factors (IGF-1 or IGF-2) to the IGF-1 receptor (IGF-1R) activates the Ras-MAPK (mitogen-activated protein kinase) and phosphoinositide 3-kinase (PI3K)/Akt pathways. Interestingly, although hepatocytes are majorly involved in the production of circulating IGF-1, IGF-1R is not expressed noticeably in normal hepatocytes [75]. However, in HCC patients, the overexpression of IGF-1R has been reported to correspond with a poor survival rate [76]. Additionally, IGF-1R signaling has been reported to promote cancer stemness properties and the epithelial–mesenchymal transition (EMT) [77].

**Figure 2:**
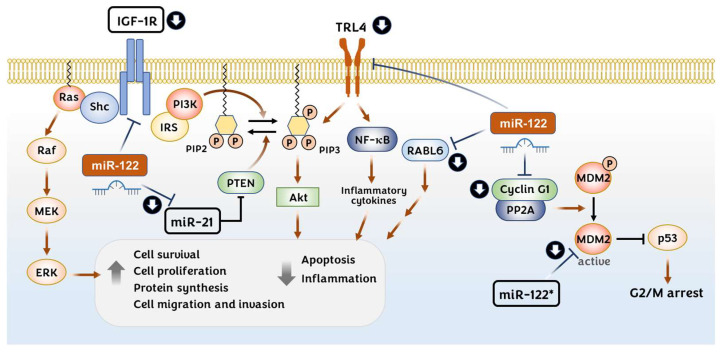
Regulation of the insulin-like growth factor 1 (IGF-1) receptor (IGF-1R); Toll-like receptor 4 (TRL4); RAB, member RAS oncogene family-like 6 (RABL6); and p53 signaling pathways by miR-122. The activated IGF-1R transduces a signal via the Ras-MAPK and PI3K/Akt pathways to enhance cell survival, proliferation, and protein synthesis while inhibiting apoptosis during hepatocellular carcinoma (HCC) progression. miR-122 suppresses the progression of HCC by directly downregulating the translation of IGF-1R and miR-21. miR-21 has an inhibitory effect on PTEN, wherein it converts PIP3 to PIP2 by dephosphorylation (Left). TRL4 can stimulates NF-κB and PI3K/Akt signaling pathways. miR-122 suppresses the expression of TRL4. Activated androgen receptor upregulates miR-122 thereby suppressing RABL6 expression (Middle). Cyclin G1/PP2A l complex activates MDM2 by dephosphorylation which promotes p53 protein degradation. miR-122*, the passenger strand, can directly downregulate the MDM2 protein, thereby upregulating p53 (Right).

IGF-1R signaling is known to be negatively regulated by miR-122 (Figure 2). IGF-1R expression has been shown to be downregulated directly by miR-122 in HCC [43,71] and breast cancer cells [78]. The protein levels of IGF-2 have been reported to increase in the miR-122 of liver KOs (LKOs), although it seems to be indirect [14]. The miR-122 expression levels correlated negatively with the IGF-1R expression levels in HCC patients and were shown to be reduced in HCC cells that were resistant to sorafenib treatment. The overexpression of miR-122 could render sorafenib-resistant cells sensitive to sorafenib by downregulating the expression of IGF-1R [71]. In this regard, IGF-1R could become a prominent target in HCC therapeutics, especially in patients with drug resistance related to aberrant IGF-1R activation.

#### 3.1.2. Cyclin G1

Various studies have suggested a role for miR-122 in the induction of G2/M phase arrest in HCC-derived cell lines by suppressing the expression of *CCNG1* (cyclin G1) [38,68,74,79,80,81]. Cyclin G1 is a noncanonical cyclin that makes a complex with Ser/Thr protein phosphatase 2A (PP2A) to activate the cyclin G1/mouse double minute 2 (Mdm2)/p53 axis. This complex dephosphorylates and activates Mdm2, leading to the degradation of the p53 protein [82]. It was shown that the downregulation of cyclin G1 by miR-122 expression stabilizes the p53 protein, leading to a reduction in the invasion capability of HCC-derived cell lines [68]. Interestingly, it was reported by Simerzin A. et al. that the passenger strand (miR-122*) targets Mdm2 directly, resulting in p53 upregulation and the induction of apoptosis in HCC cells (Figure 2). Interestingly, the inhibition of miR-122 using antagomir-122 reduced the miR-122 levels but increased the levels of miR-122* in the Huh7 cells and liver tissues of mice, resulting in the reduction of MDM2 protein levels [83]. Meanwhile, it was found that HBV infection increases cyclin G1 expression by reducing miR-122 levels [56], thereby promoting HBV replication [54]. By contrast, acute alcohol exposure increases miR-122 levels while decreasing the cyclin G1 expression in HCC cells, thereby increasing HCV replication [84].

#### 3.1.3. miR-21

miR-21 levels were found to be elevated in both the serum [59] and liver tissues [85] of HCC patients. miR-21 promotes cell migration and invasion in HCC cells by targeting a protein tyrosine phosphatase PTEN (phosphatase and tensin homolog) [85] (Figure 2). A recent meta-analysis has shown miR-21 as a biomarker for the early diagnosis of HCC [86]. Moreover, miR-21 expression is inversely correlated with that of miR-122 in HCC tissues and cells [87]. It has been shown that nuclear miR-122 binds to a 19-nt UG-containing recognition element in the basal region of pri-miR-21, thus preventing the conversion to pre-miR-21. It was further demonstrated that miR-122 retards HCC growth in a mouse xenograft model by reducing miR-21 expression [87].

#### 3.1.4. Toll-like Receptor 4 (TLR4)

Toll-like receptors (TLRs) are pattern recognition receptors of the innate immune response involved in the discrimination of pathogens, and they eventually lead to adaptive immune responses [88]. TLR4 is localized to the cell surface and recognizes the pathogen-associated molecular patterns of gram-positive bacterial products and the endogenous damage-associated molecular patterns. TLR4 expression is significantly elevated in HBV- and HCV-infected liver tissues [89,90], as well as in HCC [91,92]. It has been suggested that the activation of the TLR4 present on the surface of tumor cells promotes tumor cell survival via the activation of nuclear factor-kappa B (NF-κB) signaling and antiapoptotic proteins [93]. It has been shown that TLR4 expression is negatively correlated with miR-122 in HCC tissues and hepatocytes [94,95]. These studies indicate that miR-122 binds to the 3′-UTR of TLR4 and downregulates the expression of TLR4 in HCC cells. Furthermore, miR-122 overexpression decreases the levels of inflammatory cytokines, such as VEGF, IL-6, PEG2, Cox-2, and MMP-9, and inhibits the activities of PI3K, Akt, and NF-κB, thereby inducing apoptosis in HCC cells [94,95] (Figure 2).

#### 3.1.5. RAB, Member RAS Oncogene Family-like 6 (RABL6)

Androgen receptors suppress the invasion and migration capacities of HCC cells by upregulating miR-122. By overexpressing the androgen receptor, miR-122 could directly target the 3′-UTR of the RABL6 gene transcript, by which miR-122 suppresses HCC development in xenograft mouse models [96] (Figure 2). RABL6 has been suggested to promote cell growth and proliferation in HCC [97] and mediate the pathogenesis of HBV-related HCC. However, the mechanisms involved in the androgen receptor signal regulation of miR-122 expression and the RABL6 influence on HCC progression are still unidentified.

#### 3.1.6. MYC-Related Signaling Molecules: E2F1, TFDP2, and CLIC1

**Figure 3:**
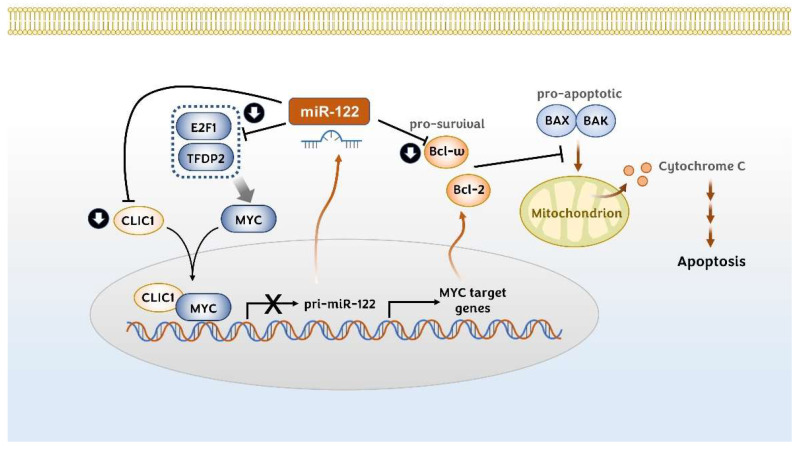
Regulation of MYC signaling pathway and Bcl family proteins by miR-122. miR-122 and MYC reciprocally regulate each other. MYC bound with CLIC1 suppresses the transcription of miR-122, whereas miR-122 downregulates CLIC1, E2F1, and TFDP2, leading to the suppression of the MYC signaling pathway. miR-122 can suppress the expression of the prosurvival Bcl family protein, Bcl-ω, and MYC-induced Bcl-2 expression, which could enhance the action of proapoptotic Bcl family members.

MYC proto-oncogene (c-Myc) is a transcriptional factor that plays an important role in the regulation of cellular growth, differentiation, and apoptosis [98,99,100]. MYC is upregulated in almost 30% of human HCC samples [101] and a meta-analysis has shown that high MYC expression indicates poor overall survival and disease-free survival in HCC patients [102]. Several studies have indicated that miR-122 can regulate MYC signaling in hepatic cells indirectly [14,103,104,105]. Chromatin immunoprecipitation and ectopic expression/knockdown assays have shown that MYC and miR-122 regulate each other through a reciprocal feedback regulation mechanism [104]. MYC inhibits the gene expression of miR-122 transcriptionally by binding with its promoter in hepatic cells. Reciprocally, miR-122 indirectly suppresses MYC transcription by targeting *E2f1* (E2F1) and *Tfdp2* (transcription factor dimerization partner 2) [104] (Figure 3). Additionally, Jiang X. et al. have shown a positive feedback regulatory circuit that exists between the intracellular chloride channel protein (CLIC1) and MYC [103]. This study showed that CLIC1 is upregulated in HCC tissues and cell lines, and that it promotes the proliferation, invasion, and migration of HCC cells in vivo and in vitro. The binding of CLIC1 with MYC enhances the transcriptional activity of MYC. Reciprocally, MYC also positively regulates CLIC1, forming a positive feedback loop. In this context, miR-122 has been suggested to target and downregulate CLIC1 in HCC cells [103].

#### 3.1.7. Bcl-w (BCL2L2)

The Bcl-2 family is divided largely into two groups: proapoptotic Bcl-2 family members such as Bak, Bax, and Bok as well as BH3-only protein members, and antiapoptotic members such as Bcl-2, Bcl-x_L_, Bcl-w, and Mcl-2. The Bcl-2 family proteins play a central role in cell death, and their expression and function contribute to the progression of cancers. For example, the elevated expression of the antiapoptotic Bcl-2 or Bcl-xL can contribute to the development of HCC [106,107].

Many studies indicate that miR-122 represses Bcl-w (*BCL2L2*) expression in various cancer cell lines, including HCC [69,79,81,108]. These studies have shown that miR-122 binds directly to the 3′-untranslated region (3′-UTR) of the Bcl-w gene, leading to the activation of caspase-3 and growth inhibition (Figure 3). It was also revealed that Bcl-2 expression is negatively correlated with miR-122. Specifically, Bcl-2 expression was highly enhanced in the miR-122 germline and LKO mice when compared with wild-type mice [105]. It was suggested that Bcl-2 is indirectly regulated via the miR-122/MYC/Bcl-2 pathway because the Bcl-2 transcript has no miR-122 seed region in the promoter region.

### 3.2. Metastasis and Epithelial–Mesenchymal Transition 

Increasing evidence has shown that miR-122 can regulate invasion and metastasis in HCC by regulating the type-3 EMT [64,65,109,110]. EMT signaling can be initiated by various EMT inducers: the transforming growth factor-beta (TGF-β), the receptor tyrosine kinase (RTK), Wnt/β-catenin, NOTCH, hedgehog, and the signal transducer and activator of transcription 3 (STAT3). These inducers activate crucial transcription factors: the Snail family members Snail/Slug, the zinc-finger E-box binding homeobox family of transcription factors ZEB1/ZEB2, and the Twist family of (bHLH) transcription factors TWIST1/TWIST2. Finally, this activation leads to the downregulation of epithelial biomarkers and the upregulation of mesenchymal biomarkers [111]. In this context, miR-122 expression suppresses the expression of various EMT inducers and mesenchymal markers (e.g., N-cadherin, vimentin, and fibronectin), while increasing the expression of epithelial markers (e.g., the cell adhesion proteins E-cadherin and BVES), both directly and indirectly.

#### 3.2.1. Wnt/β-Catenin Pathway

The aberrant activation of the Wnt/β-catenin pathway is one of the key signaling pathways that contributes to the metastatic ability in HCC. The binding of the Wnt protein to Frizzled receptors triggers β-catenin activation, which can result in the expression of the Wnt target genes. Deletions or missense mutations in the β-catenin gene (*CTNNB1*) are known to be the most common genetic abnormality related to HCC development [112].

**Figure 4:**
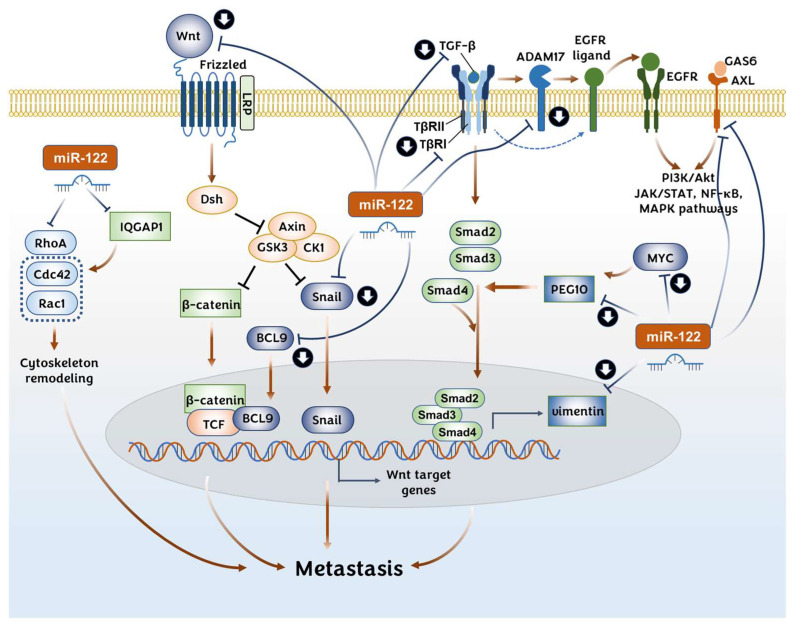
Regulation of metastasis of cancer cells by miR-122. Without any external stimulation, β-catenin is phosphorylated by the degradation complex kinases, comprising glycogen synthase kinase 3 (GSK3) and casein kinase 1α (CK1α). This leads to the degradation of β-catenin by the ubiquitin–proteasome pathway. The binding of the Wnt protein to the Frizzled receptor and co-receptors, such as the low-density lipoprotein receptor-related protein 5 (LRP5) or LRP6, initiates the dissociation of the degradation complex kinases, thereby stabilizing β-catenin and leading to the expression of Wnt target genes. miR-122 can downregulate the expression of WNT, BCL9, and vimentin, leading to the loss of their metastatic ability. The TGF-β signaling suppresses apoptosis and promotes EMT at the later stages of hepatocellular carcinoma (HCC) development. The activation of TGF-β transduces a signal through Smad proteins. Also, PEG10 regulates TGF-β signaling positively in HCC. miR-122 downregulates the expression of TGF-β, TβRI, and PEG10. TGF-β signaling can also enhance the EGFR signaling by activating ADAM17. Signaling by the AXL receptor, another target of miR-122, also contributes to the metastasis of HCC by activating PI3k/Akt, JAK/STAT, NF-κB, and MAPK pathways.

The Wnt/β-catenin signaling pathway is one of the major targets regulated by miR-122 in HCC cells [113]. Overexpressed miR-122 reduces the Wint1 expression in HCC cells by the direct regulation of the 3′-UTR of the *Wnt1* gene [45,67,114] and *Bcl9* gene [113] (Figure 4). Additionally, the expressions of β-catenin and T-cell factor 4, the downstream proteins of the Wnt signaling pathway, were reportedly reduced by miR-122 [45]. Interestingly, the study by Xu J. et al. indicated that miR-122 may regulate Wnt1 expression only at the protein level by a posttranslational regulation, but not at the mRNA level [45]. A study on Argonaute crosslinking immunoprecipitation (Ago-CLIP) sequencing using human HCC tissues and mouse miR-122 KO liver tissue discovered that the B-cell lymphoma protein 9 (BCL9), solute carrier family 52 (riboflavin transporter) member 2, and syntaxin 6 are targets of miR-122 [113]. Among them, BCL9 is a transcriptional cofactor required in the activation of the Wnt/β-catenin pathway in HCC [115] and is associated with intrahepatic metastasis and a poor prognosis [116] (Figure 4). The EMT transcription factors SNAI1 (Snail) and SNAI2 (Slug) are members of the Snail family of transcription factors that are activated via EMT induction through diverse signaling cascades, including TGF-β, RTKs, and the Wnt signaling pathway [111]. SNAI1 and SNAI2 are direct targets of miR-122 in HCC cells, as shown by the luciferase reporter assay [46].

#### 3.2.2. TGF-β Signaling Pathway

Transforming growth factor-beta (TGF-β) signaling plays a bifunctional role as both a proto-oncogene and a tumor suppressor gene in HCC development. Although still controversial, it is generally considered that TGF-β’s role is context-dependent: at the normal or early phases of HCC, TGF-β signaling suppresses the proliferation of hepatocytes, but at a later phase, it suppresses apoptosis and promotes the EMT [117,118]. The activation of TGF-β receptors transduces a signal to a series of Smad (Mothers Against Decapentaplegic homolog) proteins that transactivate diverse genes. Besides this canonical Smad pathway, TGF-β activates the epidermal growth factor receptor (EGFR) pathway by upregulating the expression of EGFR ligands and by activating the metalloprotease activity of ADAM17, which leads to the shedding of EGFR ligands [119,120] (Figure 4).

Hepatic stellate cells (HSCs) comprise a minor cell population and are located in the perisinusoidal space in the liver. These cells are activated during liver injury to protect the liver. However, sustained activation results in fibrosis, cirrhosis, and progression to HCC [121]. As TGF-β serves a critical role in the activation of HSCs, miR-122 can suppress the progress of fibrosis and EMT in HSCs by inhibiting the TGF-β signaling pathway [122,123] (Figure 4). Zeng C. et al. further showed that the intravenous injection of an miR-122-expressing lentivirus reduced the level of fibrosis markers in mouse livers treated with carbon tetrachloride [122]. Interestingly, miR-122 regulates TGF-β signaling with different mechanisms depending on the species. miR-122 suppresses the expression of *TGFβ1* in humans by targeting the 5′-UTR region, whereas it suppresses *TGFβR1* in mice by targeting the 3′-UTR region [124].

Shyu Y.C. et al. have shown that paternally expressed gene 10 (PEG10) expression is downregulated in miR-122-overexpressed HCC cells, whereas it is upregulated in the livers of miR-122 KO mice (Figure 4). The direct binding of miR-122 to the 3′-UTR of PEG10 was proven in this study [125]. PEG10 has been considered to function as a transcription factor and is expressed highly in the placenta, ovary, and testis. Generally, PEG10 promotes tumor cell proliferation and metastasis while inhibiting apoptosis in various types of cancer [126]. Interestingly, PEG10 expression is very low in liver tissue, but is overexpressed in HCC patients, implying that it may act in tumor promotion [126,127,128,129]. It seems that PEG10 may positively regulate the TGF-β signaling pathway in HCC, although the opposite role has also been reported in various cancers [126,130]. Moreover, the transcriptional regulation of PEG10 by E2F-1/-4, MYC, and the androgen receptor have been previously reported in various cancers [126,130,131].

#### 3.2.3. RTK AXL

The dysregulation of AXL signaling is associated with the development of cancers, including HCC [132]. AXL is a member of the TAM (TYRO3-AXL-MER) family of RTKs. The AXL gene was first identified in patients with chronic myelogenous leukemia and is known for its transforming activity [133]. When bound with its ligand growth arrest-specific gene 6 (GAS6), AXL promotes tumor proliferation, resistance to chemotherapy, immune suppression, and EMT through various signaling pathways such as the PI3K/Akt, JAK/STAT, NF-κB, and MAPK signaling pathways [134,135]. AXL expression is regulated positively by TLR and the vimentin-ERK axis, and is regulated negatively by the binding of miR-122 in the 3′-UTR of the AXL gene transcript (Figure 4). AXL is upregulated in miR-122 KO mouse livers at the transcription and protein levels, contributing to HCC development. However, the detailed mechanisms involved in AXL contributing to tumorigenesis have not been explored [136].

#### 3.2.4. Rho GTPases Signaling: RhoA and IQGAP1

RhoA is a small GTPase protein of the Rho family. RhoA is involved in various cellular functions, including actin organization, cell movement, the cell cycle, DNA repair, metabolism, keratinization, and vesicular transport [137]. RhoA is frequently overexpressed in HCC and is involved in an aggressive phenotype via cytoskeleton remodeling by the RhoA/ROCK pathway [138,139]. The increased protein level of RhoA was detected in miR-122 KO mouse livers [14], and along with this finding, miR-122 overexpression was found to downregulate RhoA, Rac1, and Cdc42, leading to the suppression of migration and invasion in HCC cells [64,110]. Among them, RhoA is the direct target of miR-122, as revealed by a luciferase reporter assay, and RhoA overexpression could reverse the miR-122-induced mesenchymal–epithelial transition (MET) [64,110] (Figure 4). Coulouarn C. et al. suggested Rac1 and RhoA as central regulators of cell motility from their Ingenuity pathway analysis [64].

IQGAP1 (isoleucine–glutamine motif-containing GTPase-activating protein 1) was also reported to be repressed by the overexpression of miR-122. The inhibitory effect of miR-122 on IQGAP1 was proven at the protein level, and its regulating activity on 3′-UTR of *IQGAP1* was confirmed by a luciferase activity assay in Huh 7 cells [14,20]. Analyses of rat and human HCC samples have shown that the expression of IQGAP1 and vimentin is upregulated, and that their levels are dependent on the HCC development phase [140,141,142]. The Ras GTPase-activating-like protein IQGAP1 has no GAP activity, although it has a GAP domain homologous to sar1 and it binds to the GTP-bound form of the Rho GTPases Rac1 and Cdc42, which are Rho/Rac family small GTPases [143]. It is considered a scaffold protein involved in regulating the cell cycle by organizing the actin cytoskeleton, transcription, cellular adhesion, and migration in cancer progression [144,145,146] (Figure 4).

#### 3.2.5. ADAM Metallopeptidases: ADAM10 and ADAM17

miR-122 negatively regulates ADAM10 (a disintegrin and metalloprotease family 10) and ADAM17 by interacting with their 3′-UTR [42,43,65]. ADAM metallopeptidases are single-membrane-spanning proteases consisting of 22 members that play a crucial role in the cleavage-mediated release and activation of various membrane-anchored proteins [147,148]. Both ADAM10 and ADAM17 shed a variety of molecules. For example, ADAM10 sheds cadherins and the Notch receptors [148]. ADAM10 and ADAM17 cleave AXL at its ectodomain, resulting in the release of soluble AXL (sAXL). sAXL has been known to increase in advanced fibrosis and HCC [149]. ADAM17, also called the tumor necrosis factor-α-converting enzyme, plays a crucial role in the cleavage-mediated release and activation of various membrane-anchored proteins, L-selectin [150], and EGFR ligands, such as tumor necrosis factor–alpha [151] and amphiregulin [152]. ADAM10 and ADAM17 have been known to be highly expressed in various types of cancer, including HCC, and to promote invasiveness [65,153,154,155,156,157,158].

#### 3.2.6. Mesenchymal Biomarker: Vimentin and Lamin B2

The downregulation of vimentin by miR-122 has been previously reported in HCC cells [43,65]. Recently, vimentin was demonstrated as a direct target of miR-122 by luciferase reporter analysis in Huh-7 cells [20,159]. Vimentin is a type III intermediate filament protein implicated in extracellular attachment, migration, and cell signaling. It is considered one of the key biomarkers of EMT and is upregulated during cancer metastasis [111]. In HCC, microarray analysis showed that vimentin expression was associated with the metastasis of cancer cells [160] (Figure 4).

Lamins are type V intermediate filament proteins that form the nuclear lamina in the nuclear envelope. There are different types of lamins. Lamins A and C can be formed from the *LMNA* gene via alternative splicing. Lamins B1 and B2 are produced from two different genes, *LMNB1* and *LMNB2*. Their aberrant regulation has been associated with the development and progression of HCC [161,162,163]. A recent study has shown the possible involvement of lamin B2 in the proliferation, migration, and invasion of HCC cells. miR-122 directly binds with the 3′-UTR of LMNB2 and downregulates its expression in HCC cells and in xenograft assays in vivo [20,48].

### 3.3. Epigenetic Regulators 

#### 3.3.1. Histone Lysine Methyltransferase G9a 

**Figure 5:**
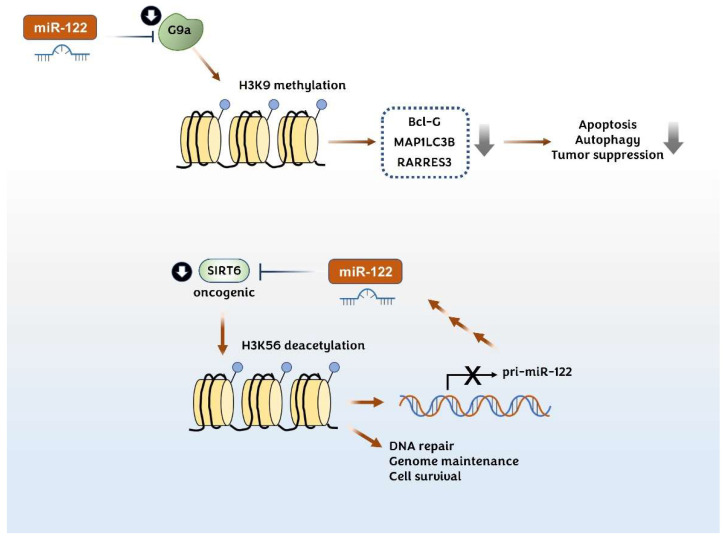
Effect of miR-122 on the epigenetic regulation of cancer cells. H3K9 methylation by G9a reduces the expression of the proapoptotic regulators Bcl-G, MAP1LC3B, and RARRES3 in hepatocellular carcinoma (HCC) (upper). H3K56 deacetylation by SIRT6 is involved in DNA repair, genome maintenance, and cell survival leading to carcinogenesis (lower). miR-122 suppresses the progression of HCC by directly downregulating G9a and SIRT6. SIRT6 and miR-122 reciprocally regulate each other via a negative feedback mechanism.

Histone lysine methyltransferase G9a (also known as euchromatic histone lysine N-methyltransferase 2, EHMT2) stimulates the formation of heterochromatin by catalyzing the mono and di-methylation of histone 3 at lysine 9 (H3K9 me2). The upregulation of G9a is involved in the progression of HCC by silencing various genes, including the proapoptotic genes, Bcl-G [164], MAP1LC3B (microtubule-associated protein light chain 3β), [165] and RARRES3 (retinoic acid receptor responder protein 3) [166]. Yuan L.T. et al. showed that the G9a expression level correlates with DNA methylation and negatively correlates with the miR-122 expression level in the TCGA HCC dataset [167]. They further showed that miR-122 directly binds to the 3′-UTR of G9a and downregulates its expression in HCC cells. G9a shRNA-mediated knockdown in a xenograft [167] and in liver-specific G9a-deficient mouse models [164] demonstrated that G9a deficiency reduces the tumorigenicity of HCC (Figure 5).

#### 3.3.2. Histone Lysine Deacetylase SIRT6 

It has been reported that miR-122 and SIRT6 negatively regulate each other’s gene expression. SIRT6 regulates the miR-122 expression by deacetylating H3K56 in the promoter region of miR-122, whereas miR-122 reduces the level of SIRT6 by binding to SIRT6 3′-UTR [73]. SIRT6 is a member of the sirtuin (SIRT) family of NAD+-dependent deacetylases. SIRT6 has deacetylase activity for several proteins, including histones H3 and H4, and also has mono(ADP-ribosyl)transferase activity over poly(ADP-ribose)polymerase 1. SIRT6 is involved in DNA repair, genome maintenance, inflammation, and metabolism (Figure 5). SIRT6 transgenic mice showed an increased lifespan [168]. Interestingly, SIRT6 functions as both a tumor suppressor and an oncogenic protein. For instance, some studies indicate that SIRT6 is upregulated in HCC tissues and cell lines and functions as a procarcinogenic protein. The loss of SIRT6 results in DNA damage-mediated G2/M phase arrest [169], increases the Bax protein expression [170], and downregulates the Bcl-2 expression and ERK1/2 activity [171]. By contrast, tumor-suppressive roles for SIRT6 in HCC models have also been reported [172,173]. Elharnanti S. et al. have shown that the patient groups showed a strong negative correlation between SIRT6 and miR-122 expression levels and showed a significantly poor survival rate in Kaplan–Meier analysis [73]. Although they have suggested that the interplay between miR-122 and SIRT6 affects the fatty acid metabolism in HCC cells, it seems that more studies are needed to elucidate the exact mechanism in HCC development.

### 3.4. Energy Metabolism 

Cancer cells are mostly dependent on glycolysis for energy production, instead of using the more efficient mitochondrial oxidative phosphorylation [174]. This reprogrammed aerobic glycolysis allows cancer cells to use glucose metabolites as precursors for the biosynthesis of the macromolecules required for cell proliferation [175]. In HCC, glycolysis and the pentose phosphate pathway (PPP) enzymes are highly expressed while gluconeogenesis and glycogenesis enzymes are generally transcriptionally suppressed. Another feature of aerobic glycolysis is that pyruvate, the final product of glycolysis, tends to be reduced into lactate instead of being oxidized into acetyl-coenzyme A (acetyl-CoA) by lactate dehydrogenase. By contrast, the metabolites and enzymes related to the TCA cycle, glycogenesis, and oxidative phosphorylation are typically inactivated and reduced in HCC [176]. A genome-scale analysis based on the metabolic networks stratified HCC into three subtypes based on the differences in metabolic and signaling pathways and the survival rate. In this regard, the activation of alternative isotypes of metabolic enzymes such as acetyl-CoA synthases (ACSS1, ACSS2, and ACSS3), aldolase (ALDOA and ALODB), pyruvate kinases (PK) (PKM and PKLR), and mitochondrial C1-tetrahydrofolate dehydrogenases (MTHFD1L/MTHFD2/MTHFD1) are critical determinants in the progression of HCC [177]. Another genome-wide gene expression profiling analysis revealed that genes related to β-oxidation, the urea cycle, and amino acid metabolism are suppressed while those of glycolysis, PPP, and fatty acid biosynthesis are upregulated in HCC [178,179]. The use of miR-122 inhibitors resulted in reduced cholesterol synthesis, reduced plasma cholesterol levels, and increased hepatic fatty acid oxidation in vivo [16,17].

#### 3.4.1. Glucose Metabolism

**Figure 6:**
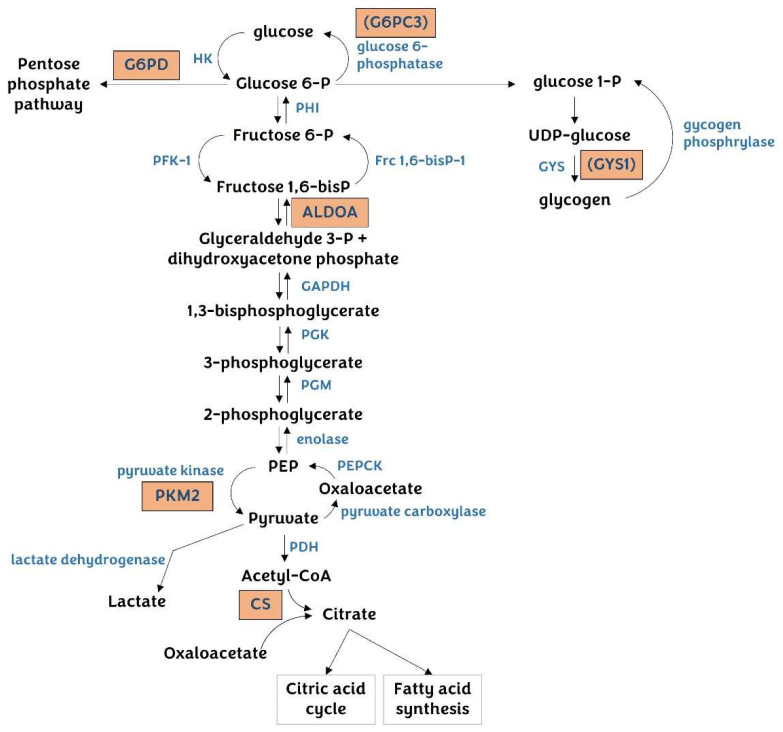
Regulation of glucose metabolism by miR-122 in hepatocellular carcinoma. miR-122 targets ALDOA and PKM2 in the glycolysis pathway. ALDOA is one of the key regulators in hepatocellular carcinoma (HCC) development, wherein its levels increase under hypoxic conditions in HCC cells. PKM2 is responsible for aerobic glycolysis, which is a prominent characteristic of cancer cells. PPP is activated in cancer cells, and G6PC is the enzyme that catalyzes the rate-determining step in the activation of PPP. CS catalyzes the biosynthesis of citrate, which is a key enzyme in fatty acid synthesis in HCC. G6PC3 is one of the isotypes of G6P that produces glucose from glucose 6-phosphate in the gluconeogenesis pathway. GYS1 mediates the synthesis of glycogen. Although G6PC3 and GYS1 are direct targets of miR-122 in HCC, the functional link of these enzymes to HCC is not clear yet.

Many miR-122 targets involved in glucose metabolism have been related to HCC, such as aldolase A (ALDOA), citrate synthase (CS), glycogen synthase 1 (GYS1), glucose 6-phosphatase catalytic subunit 3 (G6PC3), and pyruvate kinase M2 (PKM2) (Figure 6) [17,20,64,180]. ALDOA, a direct target of miR-122 [20,181,182], cleaves fructose 1,6 bisphosphate to generate dihydroxyacetone phosphate and glyceraldehyde 3-phosphate in glycolysis. Genome-wide CRISPR/CRISPR-associated 9 (Cas9) KO library screening showed ALDOA to be highly upregulated under hypoxic conditions by HIF-1α in HCC cells. A metabolite analysis of ALDOA-knockdown HepG2 cells revealed lactate to be a majorly affected metabolite. Furthermore, ALDOA-knockdown or KO HCC cells showed reduced cell proliferation and migration, especially under hypoxia, and also suppressed tumor growth and metastasis in an orthotopic xenograft mouse model [183].

PK is involved in the final step of glycolysis, which is the conversion of phosphoenolpyruvate into pyruvate. There are four PK isotypes: the PKL (liver) and PKR (red blood cell) isoforms encoded by the *PKLR* gene, and the PKM1 and PKM2 muscle isoforms encoded by the *PKM* gene [184,185]. The four PK isoforms form active tetramers, whereas PKM2 forms less active dimers, which promotes aerobic glycolysis in which pyruvate is majorly converted to lactate through lactate dehydrogenase. Many studies have shown that PKM2 is dominantly expressed in cancer cells and promotes the anabolic synthesis of the macromolecules required for cell proliferation through PPP [186]. PKM2 is upregulated in HCC patients and shows a positive correlation with poor prognosis, possibly sensitizing HCC to immune checkpoint blockade [187]. The direct interaction of miR-122 with the PKM gene’s 3′-UTR region has been shown in HCC cells by luciferase assays and western blotting. We also confirmed the direct regulation of the PKM gene by miR-122 (unpublished data). The expression of miR-122 reduces lactate production and increases oxygen consumption by suppressing PKM2 expression, indicating the shift from glycolysis to oxidative phosphorylation [20,47,72,180,188].

CS, GYS1, and G6PC3 were identified as miR-122 targets by a luciferase reporter assay [20]. CS catalyzes the biosynthesis of citrate from acetyl-CoA and oxaloacetate in the mitochondria, which is the first and rate-limiting step of the TCA cycle. Also, citrate is used as a shuttle for acetyl groups to be used for the biosynthesis of fatty acids in the cytosol. The level of CS or enzyme activity is significantly increased in human and mouse models of HCC [178,189].

Glycogen synthase (GYS) and glucose 6-phosphatase (G6P) are enzymes required for glycogen synthesis and gluconeogenesis, respectively. miR-122 suppresses muscle isoform GYS1 expression by binding to three miR-122 target sites in the 3′-UTR. However, the liver-specific isoform GYS2 lacks miR-122 target sites [20]. Additionally, glycogen synthesis is typically suppressed in HCC. Meanwhile, glucose 6-phosphatase (G6P) converts glucose 6-phosphate to glucose in the gluconeogenesis pathway and is encoded by G6PC1, G6PC2, and G6PC3. G6PC1 is majorly present in the liver tissues, whereas G6PC3 is ubiquitously expressed. It has been reported that G6PC3 deficiency causes congenital neutropenia [190]. It seems that the relationship between G6PC1 and HCC has not yet been established. Altogether, GYS1 and G6PC3 may not be the key regulatory targets of miR-122 in terms of HCC development.

Glucose 6-phosphate dehydrogenase (G6PD) is the rate-limiting enzyme in the PPP, providing precursors for the biosynthesis of nucleotides and lipids, and is known to be activated in carcinogenesis. Analyses of RNA-seq data and clinical data from TCGA have shown that the G6PD levels correlate with tumor grade, tumor recurrence, and poor patient survival in HCC [191]. In this context, overexpressed miR-122 targets G6PD by binding to the 3′-UTR and represses PPP, leading to reduced HCC cell viability.

#### 3.4.2. Lipid Metabolism

Genome-wide gene expression profile analyses showed that β-oxidation is suppressed while fatty acid biosynthesis is upregulated in HCC [178,179]. Accordingly, the use of miR-122 inhibitors resulted in reduced cholesterol synthesis, reduced plasma cholesterol levels, and increased hepatic fatty acid oxidation in vivo [16,17].

The study performed by Elhanati S. et al. suggests that miR-122 and SIRT6 negatively regulate each other. Both factors co-regulate fatty acid metabolism, and the dysregulation in the balance of reciprocal regulation is often correlated with HCC progression. They have also shown that the β-oxidation-related genes *Hadhb*, *Cpt1*, *Crot*, and *Acly* are oppositely regulated by miR-122 and SIRT6 in HCC cells [73]. An enrichment assay performed by Burchard J. et al. indicated that a set of metabolic genes, which code for mitochondrially localized proteins and fatty acid and amino acid metabolic functions, are the secondary targets positively correlated with miR-122. They suggested that the *PPARGC1A* (PPARG Coactivator 1 Alpha, PGC-1α) gene is the key regulator of mitochondrial biogenesis related to the metabolic function of miR-122 [44]. Unfortunately, the detailed mechanisms were not mentioned in these studies.

The transcription factor cut-like homeobox 1 (CUTL1) protein is a direct target of miR-122, which is related to liver development. As proven by ectopic expression and knockdown studies, miR-122 can promote terminal differentiation by repressing CUTL1 expression. In this study, the cholesterol-7α hydroxylase gene (*CYP7A1*) was suggested as one of the responsible targets of CUTL1 [192]. However, the mechanism involved in the differentiation of CUTL1 to the liver phenotype has not been well described.

### 3.5. Upstream Regulators of miR-122 in HCC

#### 3.5.1. Epigenetic Regulation

**Figure 7:**
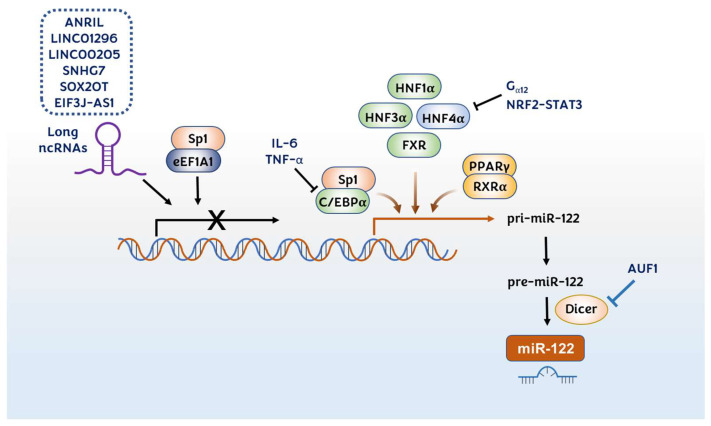
Upstream regulators of miR-122 in hepatocellular carcinoma. Long ncRNAs such as ANRIL, LINC01296, LINC00205, SNHG7, SOX2OT, and EIF3J-AS1 downregulate the transcription of miR-122. LETFs such as HNF1α, HNF3α, HNF3β, HNF4α, and C/EBPα, as well as PPARγ/RXRα and FXR, positively regulate miR-122 transcription by binding to the promoter region of the miR-122 gene in the liver. Sp1 has a dual role in miR-122 regulation. When Sp1 makes a complex with C/EBPα, it promotes miR-122 transcription, whereas the complex of Sp1 and eEF1A1 suppresses miR-122 transcription. HNF4α is regulated by G_α12_ and NRF2–STAT3 negatively. miR-122 can be regulated at the maturation step positively by Dicer1 and negatively by AUF1.

Numerous studies have shown that liver-enriched transcription factors (LETFs)—hepatocyte nuclear factor (HNF) 1α, HNF3α, HNF3β, HNF4α, and CCAAT/enhancer-binding protein (C/EBP) α—are upstream regulators of miR-122 (Figure 7). Overexpression and knockdown studies have shown that LETFs can elevate miR-122 expression in the liver of developing mouse embryos and human HCC cell lines [64,192,193]. The regulation of miR-122 by these LETFs affects the viability, motility, and invasion of HCC cells via the downregulation of various downstream effectors such as the RhoA/ROCK pathway [110] and ADAM17 [42]. The correlation between miR-122 and HNF4α was additionally confirmed during the hepatocytic differentiation process of embryonic stem cells [194] and iron overload-mediated hepatic inflammation [195]. The transcriptional regulation sites within the miR-122 promoter region were confirmed by the luciferase reporter systems [192,196] and the Hnf4a liver-specific mouse model [197].

HNF4α itself has been shown to be regulated by G_α12_ and the NRF2–STAT3 signaling pathway [198,199] (Figure 7). G_α12_ destabilizes HNF4α by stimulating ubiquitination. Thus, G_α12_ knockdown increases miR-122 expression and decreases c-MET expression, thus resulting in tumor cell apoptosis [198]. It was also shown that persistent HCV infection activates the NRF2–STAT3 signaling pathway, which suppresses HNF4α expression as a cytoprotective response (Figure 7). Accordingly, the activation of the NRF2–STAT3 signaling pathway leads to the reduction of miR-122 in persistent HCV infection [199]. Additionally, miR-122 can also positively regulate HNF4A expression, constituting the miR-122/FoxA1/HNF4α-positive feedback loop [194].

A lowered C/EBPα expression has been reported in HCC [200,201]. Zeng C. et al. have shown that C/EBPα transactivates the miR-122 gene by directly binding to at least two different sites on its promoter, indicating that the reduction in C/EBPα activity leads to the downregulation of miR-122 in HCC [201] (Figure 7). Additionally, this group showed that C/EBPα transactivates the miR-122 gene by cooperating with another transcription factor, Sp1 [202]. They have shown that Sp1 and C/EBPα bind to the positive regulatory site D in the miR-122 promoter. Contrarily, when Sp1 binds with the eukaryotic translation elongation factors 1 alpha 1 (eEF1A1), they bind to the negative regulatory site E and suppress the miR-122 expression (Figure 7). In HCC tissues, the Sp1 and eEF1A1 expressions were enhanced, whereas the C/EBPα and miR-122 expressions were reduced [202]. Moreover, it has been reported that the IL-6 and TNF-α levels were elevated and the miR-122 levels were decreased in mouse and rat models of diethylnitrosamine-induced HCC [55]. IL-6 and TNF-α suppressed miR-122, by both directly downregulating the transcription factor C/EBPα and indirectly upregulating MYC, which blocks C/EBPα-mediated miR-122 transcription.

The peroxisome proliferator-activated receptor-gamma (PPARγ)/retinoid X receptor alpha (RXRα) complex transactivates the miR-122 expression by binding to DR1 and DR2 motif sites of the miR-122 promoter region (Figure 7). 5′aza-2′deoxycytidine or 4-phenyl butyric acid treatment increases the association of PPARγ/RXRα and decreases the association of corepressors, NCoR (nuclear receptor corepressor) and SMRT (silencing mediator of retinoic acid and thyroid hormone receptor), in these sites in HCC cells [203]. The farnesoid X receptor (FXR) is a nuclear receptor activated by bile acids; it acts as a transcription factor that plays a protective role against liver carcinogenesis [204]. He J. et al. have shown that FXR expression is positively correlated with that of miR-122 in HCC tissues. This study showed that FXR is a transcriptional activator that directly binds to the DR2 element (−338 to −325) in the miR-122 promoter region, thereby suppressing the proliferation of HCC cells and the growth of HCC xenografts in vivo [205].

#### 3.5.2. Regulation by Long ncRNAs

Recently, long ncRNAs (lncRNAs) have been suggested as major factors involved in the progression of HCC, which affect viral infection, regenerative signals, and the hypoxic microenvironment [206]. Several lncRNAs have been introduced as upstream regulators for miR-122 expression. Antisense noncoding RNAs in the INK4 locus (ANRIL) [207], LINC01296 [208], LINC00205 [209], small nucleolar RNA host gene 7 (SNHG7) [210], SOX2OT [211], and EIF3J-AS1 [212] have been identified to be upregulated in HCC tissues and to be negatively associated with miR-122 expression (Figure 7). It was found that miR-122 contained a sequence complementary to these lncRNAs; thus, these lncRNAs could downregulate the miR-122 expression, resulting in the promotion of proliferation, migration, and invasion of HCC cells.

#### 3.5.3. Regulation in Maturation Process

miR-122 is also regulated during the miRNA maturation process. Dicer is a type III RNase that is involved in the maturation of double-stranded precursor microRNAs (pre-miRNAs). Wu X. et al. reported that Dicer1 and AUF1 are reciprocally regulated in both human HCC tissues and cell lines, and that Dicer1 expression is negatively regulated by the AU-rich element-binding factor AUF1 [213]. By suppressing the expression of Dicer1, AUF1 downregulates the maturation of miR-122, which further contributes to the development of HCC (Figure 7).

## 4. Perspectives 

The dysregulation of miR-122 is an important factor in the progression of HCC. Current data indicates that miR-122 targets are involved in various cellular functions such as cell growth, survival, cell death, EMT, and metabolism. Interestingly, attempts have been made in the past to reduce the miR-122 levels to treat HCV infection and relieve symptoms related to abnormal lipid metabolism. A clinical trial targeting miR-122 with anti-miR-122 oligonucleotides—Miravirsen, the first miRNA-targeted drug—has been initiated for the treatment of HCV infection [33]. The HCV life cycle is also intimately linked to lipid and cholesterol metabolism, as products of the cholesterol biosynthetic pathway are essential host factors for HCV replication [30]. However, for the treatment of fibrosis, cirrhosis, and HCC, the opposite strategy using the miR-122 mimic or agonists can be applied to elevate the miR-122 levels in the liver.

miR-122 regulates the development of HCC through several physiological aspects. Functionally, miR-122 is involved in diverse signaling pathways related to cell proliferation, death, metastasis, and metabolism, which altogether show anticarcinogenic effects in HCC. Metabolic alterations are especially notable in HCC, and controlling the metabolic activity may provide alternative advantages in the treatment of HCC along with cotreatment with chemotherapeutic drugs. Unfortunately, compared with glucose metabolism, the relevant miR-122 targets in lipid metabolism have been less explored in terms of HCC. Thus, for a better understanding of miR-122-based treatment, it is necessary to explore more targets in lipid metabolism in the future.

One of the prominent features of HCC is the heterogeneity of cancer types, which is closely related to the diverse metabolic state of HCC patients. This implies that, depending on the metabolic state of the individuals, different treatment strategies can be applied.

This review suggests that miR-122 can be a useful biomarker for diagnosis and can be developed as a promising antitumor agent for both monotherapy and combination therapies.

## References

[1] Ferlay J., Ervik M., Lam F., Colombet M., Mery L., Piñeros M., Znaor A., Soerjomataram I., Bray F. Cancer Today (Powered by GLOBOCAN 2018). https://publications.iarc.fr/577.

[2] Bray F., Ferlay J., Soerjomataram I., Siegel R.L., Torre L.A., Jemal A. (2018). Global cancer statistics 2018: GLOBOCAN estimates of incidence and mortality worldwide for 36 cancers in 185 countries. CA Cancer J. Clin..

[3] Ng J., Wu J. (2012). Hepatitis B- and hepatitis C-related hepatocellular carcinomas in the United States: Similarities and differences. Hepat. Mon..

[4] American Cancer Society (2012). Cancer Facts & Figures 2012.

[5] Lee R.C., Feinbaum R.L., Ambros V. (1993). The C. elegans heterochronic gene lin-4 encodes small RNAs with antisense complementarity to lin-14. Cell.

[6] miRBase miRBase: The microRNA Database. https://www.mirbase.org/.

[7] Bartel D.P. (2004). MicroRNAs: Genomics, biogenesis, mechanism, and function. Cell.

[8] Kiriakidou M., Nelson P.T., Kouranov A., Fitziev P., Bouyioukos C., Mourelatos Z., Hatzigeorgiou A. (2004). A combined computational-experimental approach predicts human microRNA targets. Genes Dev..

[9] Ye W., Lv Q., Wong C.K., Hu S., Fu C., Hua Z., Cai G., Li G., Yang B.B., Zhang Y. (2008). The effect of central loops in miRNA:MRE duplexes on the efficiency of miRNA-mediated gene regulation. PLoS ONE.

[10] Friedman R.C., Farh K.K., Burge C.B., Bartel D.P. (2009). Most mammalian mRNAs are conserved targets of microRNAs. Genome Res..

[11] Jopling C. (2012). Liver-specific microRNA-122: Biogenesis and function. RNA Biol..

[12] Lagos-Quintana M., Rauhut R., Yalcin A., Meyer J., Lendeckel W., Tuschl T. (2002). Identification of tissue-specific microRNAs from mouse. Curr. Biol..

[13] Etiemble J., Moroy T., Jacquemin E., Tiollais P., Buendia M.A. (1989). Fused transcripts of c-myc and a new cellular locus, hcr in a primary liver tumor. Oncogene.

[14] Hsu S.H., Wang B., Kota J., Yu J., Costinean S., Kutay H., Yu L., Bai S., La Perle K., Chivukula R.R. (2012). Essential metabolic, anti-inflammatory, and anti-tumorigenic functions of miR-122 in liver. J. Clin. Investig..

[15] Tsai W.C., Hsu S.D., Hsu C.S., Lai T.C., Chen S.J., Shen R., Huang Y., Chen H.C., Lee C.H., Tsai T.F. (2012). MicroRNA-122 plays a critical role in liver homeostasis and hepatocarcinogenesis. J. Clin. Investig..

[16] Krutzfeldt J., Rajewsky N., Braich R., Rajeev K.G., Tuschl T., Manoharan M., Stoffel M. (2005). Silencing of microRNAs in vivo with ‘antagomirs’. Nature.

[17] Esau C., Davis S., Murray S.F., Yu X.X., Pandey S.K., Pear M., Watts L., Booten S.L., Graham M., McKay R. (2006). miR-122 regulation of lipid metabolism revealed by in vivo antisense targeting. Cell Metab..

[18] Elmen J., Lindow M., Schutz S., Lawrence M., Petri A., Obad S., Lindholm M., Hedtjarn M., Hansen H.F., Berger U. (2008). LNA-mediated microRNA silencing in non-human primates. Nature.

[19] Wen J., Friedman J.R. (2012). miR-122 regulates hepatic lipid metabolism and tumor suppression. J. Clin. Investig..

[20] Boutz D.R., Collins P.J., Suresh U., Lu M., Ramirez C.M., Fernandez-Hernando C., Huang Y., Abreu Rde S., Le S.Y., Shapiro B.A. (2011). Two-tiered approach identifies a network of cancer and liver disease-related genes regulated by miR-122. J. Biol. Chem..

[21] Koberle V., Waidmann O., Kronenberger B., Andrei A., Susser S., Fuller C., Perner D., Zeuzem S., Sarrazin C., Piiper A. (2013). Serum microRNA-122 kinetics in patients with chronic hepatitis C virus infection during antiviral therapy. J. Viral Hepat..

[22] Van der Meer A.J., Farid W.R., Sonneveld M.J., de Ruiter P.E., Boonstra A., van Vuuren A.J., Verheij J., Hansen B.E., de Knegt R.J., van der Laan L.J. (2013). Sensitive detection of hepatocellular injury in chronic hepatitis C patients with circulating hepatocyte-derived microRNA-122. J. Viral Hepat..

[23] Cermelli S., Ruggieri A., Marrero J.A., Ioannou G.N., Beretta L. (2011). Circulating microRNAs in patients with chronic hepatitis C and non-alcoholic fatty liver disease. PLoS ONE.

[24] Waidmann O., Bihrer V., Pleli T., Farnik H., Berger A., Zeuzem S., Kronenberger B., Piiper A. (2012). Serum microRNA-122 levels in different groups of patients with chronic hepatitis B virus infection. J. Viral Hepat..

[25] Bandiera S., Pfeffer S., Baumert T.F., Zeisel M.B. (2015). miR-122—A key factor and therapeutic target in liver disease. J. Hepatol..

[26] Yamada H., Suzuki K., Ichino N., Ando Y., Sawada A., Osakabe K., Sugimoto K., Ohashi K., Teradaira R., Inoue T. (2013). Associations between circulating microRNAs (miR-21, miR-34a, miR-122 and miR-451) and non-alcoholic fatty liver. Clin. Chim. Acta.

[27] Miyaaki H., Ichikawa T., Kamo Y., Taura N., Honda T., Shibata H., Milazzo M., Fornari F., Gramantieri L., Bolondi L. (2014). Significance of serum and hepatic microRNA-122 levels in patients with non-alcoholic fatty liver disease. Liver Int..

[28] Long J.K., Dai W., Zheng Y.W., Zhao S.P. (2019). miR-122 promotes hepatic lipogenesis via inhibiting the LKB1/AMPK pathway by targeting Sirt1 in non-alcoholic fatty liver disease. Mol. Med..

[29] Hu Y., Peng X., Du G., Zhang Z., Zhai Y., Xiong X., Luo X. (2022). MicroRNA-122-5p Inhibition Improves Inflammation and Oxidative Stress Damage in Dietary-Induced Non-alcoholic Fatty Liver Disease Through Targeting FOXO3. Front. Physiol..

[30] Jangra R.K., Yi M., Lemon S.M. (2010). Regulation of hepatitis C virus translation and infectious virus production by the microRNA miR-122. J. Virol..

[31] Wilson J.A., Zhang C., Huys A., Richardson C.D. (2011). Human Ago2 is required for efficient microRNA 122 regulation of hepatitis C virus RNA accumulation and translation. J. Virol..

[32] Shimakami T., Yamane D., Jangra R.K., Kempf B.J., Spaniel C., Barton D.J., Lemon S.M. (2012). Stabilization of hepatitis C virus RNA by an Ago2-miR-122 complex. Proc. Natl. Acad. Sci. USA.

[33] Janssen H.L., Reesink H.W., Lawitz E.J., Zeuzem S., Rodriguez-Torres M., Patel K., van der Meer A.J., Patick A.K., Chen A., Zhou Y. (2013). Treatment of HCV infection by targeting microRNA. N. Engl. J. Med..

[34] Elemeery M.N., Mohamed M.A., Madkour M.A., Shamseya M.M., Issa N.M., Badr A.N., Ghareeb D.A., Pan C.H. (2019). MicroRNA signature in patients with hepatocellular carcinoma associated with type 2 diabetes. World J. Gastroenterol..

[35] Deng P., Li M., Wu Y. (2022). The Predictive Efficacy of Serum Exosomal microRNA-122 and microRNA-148a for Hepatocellular Carcinoma Based on Smart Healthcare. J. Healthc. Eng..

[36] Yu G., Chen X., Chen S., Ye W., Hou K., Liang M. (2016). MiR-19a, miR-122 and miR-223 are differentially regulated by hepatitis B virus X protein and involve in cell proliferation in hepatoma cells. J. Transl. Med..

[37] Li W., Xie L., He X., Li J., Tu K., Wei L., Wu J., Guo Y., Ma X., Zhang P. (2008). Diagnostic and prognostic implications of microRNAs in human hepatocellular carcinoma. Int. J. Cancer.

[38] Gramantieri L., Ferracin M., Fornari F., Veronese A., Sabbioni S., Liu C.G., Calin G.A., Giovannini C., Ferrazzi E., Grazi G.L. (2007). Cyclin G1 is a target of miR-122a, a microRNA frequently down-regulated in human hepatocellular carcinoma. Cancer Res..

[39] Kutay H., Bai S., Datta J., Motiwala T., Pogribny I., Frankel W., Jacob S.T., Ghoshal K. (2006). Downregulation of miR-122 in the rodent and human hepatocellular carcinomas. J. Cell Biochem..

[40] Thurnherr T., Mah W.C., Lei Z., Jin Y., Rozen S.G., Lee C.G. (2016). Differentially Expressed miRNAs in Hepatocellular Carcinoma Target Genes in the Genetic Information Processing and Metabolism Pathways. Sci. Rep..

[41] Ha S.Y., Yu J.I., Choi C., Kang S.Y., Joh J.W., Paik S.W., Kim S., Kim M., Park H.C., Park C.K. (2019). Prognostic significance of miR-122 expression after curative resection in patients with hepatocellular carcinoma. Sci. Rep..

[42] Yang G., Zhang M., Zhao Y., Pan Y., Kan M., Li J., He K., Zhang X. (2020). HNF-4alpha inhibits hepatocellular carcinoma cell proliferation through mir-122-adam17 pathway. PLoS ONE.

[43] Bai S., Nasser M.W., Wang B., Hsu S.H., Datta J., Kutay H., Yadav A., Nuovo G., Kumar P., Ghoshal K. (2009). MicroRNA-122 inhibits tumorigenic properties of hepatocellular carcinoma cells and sensitizes these cells to sorafenib. J. Biol. Chem..

[44] Burchard J., Zhang C., Liu A.M., Poon R.T., Lee N.P., Wong K.F., Sham P.C., Lam B.Y., Ferguson M.D., Tokiwa G. (2010). microRNA-122 as a regulator of mitochondrial metabolic gene network in hepatocellular carcinoma. Mol. Syst. Biol..

[45] Xu J., Zhu X., Wu L., Yang R., Yang Z., Wang Q., Wu F. (2012). MicroRNA-122 suppresses cell proliferation and induces cell apoptosis in hepatocellular carcinoma by directly targeting Wnt/beta-catenin pathway. Liver Int..

[46] Jin Y., Wang J., Han J., Luo D., Sun Z. (2017). MiR-122 inhibits epithelial-mesenchymal transition in hepatocellular carcinoma by targeting Snail1 and Snail2 and suppressing WNT/beta-cadherin signaling pathway. Exp. Cell Res..

[47] Xu Q., Zhang M., Tu J., Pang L., Cai W., Liu X. (2015). MicroRNA-122 affects cell aggressiveness and apoptosis by targeting PKM2 in human hepatocellular carcinoma. Oncol. Rep..

[48] Li X.N., Yang H., Yang T. (2020). miR-122 Inhibits Hepatocarcinoma Cell Progression by Targeting LMNB2. Oncol. Res..

[49] Wen D.Y., Huang J.C., Wang J.Y., Pan W.Y., Zeng J.H., Pang Y.Y., Yang H. (2018). Potential clinical value and putative biological function of miR-122-5p in hepatocellular carcinoma: A comprehensive study using microarray and RNA sequencing data. Oncol. Lett..

[50] Zhou J., Yu L., Gao X., Hu J., Wang J., Dai Z., Wang J.F., Zhang Z., Lu S., Huang X. (2011). Plasma microRNA panel to diagnose hepatitis B virus-related hepatocellular carcinoma. J. Clin. Oncol..

[51] Tan Y., Pan T., Ye Y., Ge G., Chen L., Wen D., Zou S. (2014). Serum microRNAs as potential biomarkers of primary biliary cirrhosis. PLoS ONE.

[52] Xu J., An P., Winkler C.A., Yu Y. (2020). Dysregulated microRNAs in Hepatitis B Virus-Related Hepatocellular Carcinoma: Potential as Biomarkers and Therapeutic Targets. Front. Oncol..

[53] Mahmoudian-Sani M.R., Asgharzade S., Alghasi A., Saeedi-Boroujeni A., Adnani Sadati S.J., Moradi M.T. (2019). MicroRNA-122 in patients with hepatitis B and hepatitis B virus-associated hepatocellular carcinoma. J. Gastrointest. Oncol..

[54] Wang S., Qiu L., Yan X., Jin W., Wang Y., Chen L., Wu E., Ye X., Gao G.F., Wang F. (2012). Loss of microRNA 122 expression in patients with hepatitis B enhances hepatitis B virus replication through cyclin G(1) -modulated P53 activity. Hepatology.

[55] Li C., Deng M., Hu J., Li X., Chen L., Ju Y., Hao J., Meng S. (2016). Chronic inflammation contributes to the development of hepatocellular carcinoma by decreasing miR-122 levels. Oncotarget.

[56] Li C., Wang Y., Wang S., Wu B., Hao J., Fan H., Ju Y., Ding Y., Chen L., Chu X. (2013). Hepatitis B virus mRNA-mediated miR-122 inhibition upregulates PTTG1-binding protein, which promotes hepatocellular carcinoma tumor growth and cell invasion. J. Virol..

[57] Zhao X.F., Li N., Lin D.D., Sun L.B. (2020). Circulating MicroRNA-122 for the Diagnosis of Hepatocellular Carcinoma: A Meta-Analysis. Biomed. Res. Int..

[58] Wei X.Y., Ding J., Tian W.G., Yu Y.C. (2020). MicroRNA-122 as a diagnostic biomarker for hepatocellular carcinoma related to hepatitis C virus: A meta-analysis and systematic review. J. Int. Med. Res..

[59] Xu J., Wu C., Che X., Wang L., Yu D., Zhang T., Huang L., Li H., Tan W., Wang C. (2011). Circulating microRNAs, miR-21, miR-122, and miR-223, in patients with hepatocellular carcinoma or chronic hepatitis. Mol. Carcinog..

[60] Qi P., Cheng S.Q., Wang H., Li N., Chen Y.F., Gao C.F. (2011). Serum microRNAs as biomarkers for hepatocellular carcinoma in Chinese patients with chronic hepatitis B virus infection. PLoS ONE.

[61] Bharali D., Banerjee B.D., Bharadwaj M., Husain S.A., Kar P. (2019). Expression Analysis of MicroRNA-21 and MicroRNA-122 in Hepatocellular Carcinoma. J. Clin. Exp. Hepatol..

[62] Jin Y., Wong Y.S., Goh B.K.P., Chan C.Y., Cheow P.C., Chow P.K.H., Lim T.K.H., Goh G.B.B., Krishnamoorthy T.L., Kumar R. (2019). Circulating microRNAs as Potential Diagnostic and Prognostic Biomarkers in Hepatocellular Carcinoma. Sci. Rep..

[63] Karakatsanis A., Papaconstantinou I., Gazouli M., Lyberopoulou A., Polymeneas G., Voros D. (2013). Expression of microRNAs, miR-21, miR-31, miR-122, miR-145, miR-146a, miR-200c, miR-221, miR-222, and miR-223 in patients with hepatocellular carcinoma or intrahepatic cholangiocarcinoma and its prognostic significance. Mol. Carcinog..

[64] Coulouarn C., Factor V.M., Andersen J.B., Durkin M.E., Thorgeirsson S.S. (2009). Loss of miR-122 expression in liver cancer correlates with suppression of the hepatic phenotype and gain of metastatic properties. Oncogene.

[65] Tsai W.C., Hsu P.W., Lai T.C., Chau G.Y., Lin C.W., Chen C.M., Lin C.D., Liao Y.L., Wang J.L., Chau Y.P. (2009). MicroRNA-122, a tumor suppressor microRNA that regulates intrahepatic metastasis of hepatocellular carcinoma. Hepatology.

[66] Chen C.L., Wu J.C., Chen G.Y., Yuan P.H., Tseng Y.W., Li K.C., Hwang S.M., Hu Y.C. (2015). Baculovirus-Mediated miRNA Regulation to Suppress Hepatocellular Carcinoma Tumorigenicity and Metastasis. Mol. Ther..

[67] Wang N., Wang Q., Shen D., Sun X., Cao X., Wu D. (2016). Downregulation of microRNA-122 promotes proliferation, migration, and invasion of human hepatocellular carcinoma cells by activating epithelial-mesenchymal transition. Onco. Targets Ther..

[68] Fornari F., Gramantieri L., Giovannini C., Veronese A., Ferracin M., Sabbioni S., Calin G.A., Grazi G.L., Croce C.M., Tavolari S. (2009). MiR-122/cyclin G1 interaction modulates p53 activity and affects doxorubicin sensitivity of human hepatocarcinoma cells. Cancer Res..

[69] Xu Y., Xia F., Ma L., Shan J., Shen J., Yang Z., Liu J., Cui Y., Bian X., Bie P. (2011). MicroRNA-122 sensitizes HCC cancer cells to adriamycin and vincristine through modulating expression of MDR and inducing cell cycle arrest. Cancer Lett..

[70] Cao F., Yin L.X. (2019). miR-122 enhances sensitivity of hepatocellular carcinoma to oxaliplatin via inhibiting MDR1 by targeting Wnt/beta-catenin pathway. Exp. Mol. Pathol..

[71] Xu Y., Huang J., Ma L., Shan J., Shen J., Yang Z., Liu L., Luo Y., Yao C., Qian C. (2016). MicroRNA-122 confers sorafenib resistance to hepatocellular carcinoma cells by targeting IGF-1R to regulate RAS/RAF/ERK signaling pathways. Cancer Lett..

[72] Pan C., Wang X., Shi K., Zheng Y., Li J., Chen Y., Jin L., Pan Z. (2016). MiR-122 Reverses the Doxorubicin-Resistance in Hepatocellular Carcinoma Cells through Regulating the Tumor Metabolism. PLoS ONE.

[73] Elhanati S., Ben-Hamo R., Kanfi Y., Varvak A., Glazz R., Lerrer B., Efroni S., Cohen H.Y. (2016). Reciprocal Regulation between SIRT6 and miR-122 Controls Liver Metabolism and Predicts Hepatocarcinoma Prognosis. Cell Rep..

[74] Xu G., Bu S., Wang X., Ge H. (2022). MiR-122 radiosensitize hepatocellular carcinoma cells by suppressing cyclin G1. Int. J. Radiat. Biol..

[75] Kineman R.D., Del Rio-Moreno M., Sarmento-Cabral A. (2018). 40 YEARS of IGF1: Understanding the tissue-specific roles of IGF1/IGF1R in regulating metabolism using the Cre/loxP system. J. Mol. Endocrinol..

[76] Lin S.B., Zhou L., Liang Z.Y., Zhou W.X., Jin Y. (2017). Expression of GRK2 and IGF1R in hepatocellular carcinoma: Clinicopathological and prognostic significance. J. Clin. Pathol..

[77] Ngo M.T., Jeng H.Y., Kuo Y.C., Diony Nanda J., Brahmadhi A., Ling T.Y., Chang T.S., Huang Y.H. (2021). The Role of IGF/IGF-1R Signaling in Hepatocellular Carcinomas: Stemness-Related Properties and Drug Resistance. Int. J. Mol. Sci..

[78] Wang B., Wang H., Yang Z. (2012). MiR-122 inhibits cell proliferation and tumorigenesis of breast cancer by targeting IGF1R. PLoS ONE.

[79] Ma L., Liu J., Shen J., Liu L., Wu J., Li W., Luo J., Chen Q., Qian C. (2010). Expression of miR-122 mediated by adenoviral vector induces apoptosis and cell cycle arrest of cancer cells. Cancer Biol. Ther..

[80] Ma J., Wu Q., Zhang Y., Li J., Yu Y., Pan Q., Sun F. (2014). MicroRNA sponge blocks the tumor-suppressing functions of microRNA-122 in human hepatoma and osteosarcoma cells. Oncol. Rep..

[81] Lin C.J., Gong H.Y., Tseng H.C., Wang W.L., Wu J.L. (2008). miR-122 targets an anti-apoptotic gene, Bcl-w, in human hepatocellular carcinoma cell lines. Biochem. Biophys. Res. Commun..

[82] Gordon E.M., Ravicz J.R., Liu S., Chawla S.P., Hall F.L. (2018). Cell cycle checkpoint control: The cyclin G1/Mdm2/p53 axis emerges as a strategic target for broad-spectrum cancer gene therapy—A review of molecular mechanisms for oncologists. Mol. Clin. Oncol..

[83] Simerzin A., Zorde-Khvalevsky E., Rivkin M., Adar R., Zucman-Rossi J., Couchy G., Roskams T., Govaere O., Oren M., Giladi H. (2016). The liver-specific microRNA-122*, the complementary strand of microRNA-122, acts as a tumor suppressor by modulating the p53/mouse double minute 2 homolog circuitry. Hepatology.

[84] Hou W., Bukong T.N., Kodys K., Szabo G. (2013). Alcohol facilitates HCV RNA replication via up-regulation of miR-122 expression and inhibition of cyclin G1 in human hepatoma cells. Alcohol. Clin. Exp. Res..

[85] Meng F., Henson R., Wehbe-Janek H., Ghoshal K., Jacob S.T., Patel T. (2007). MicroRNA-21 regulates expression of the PTEN tumor suppressor gene in human hepatocellular cancer. Gastroenterology.

[86] Qu J., Yang J., Chen M., Cui L., Wang T., Gao W., Tian J., Wei R. (2019). MicroRNA-21 as a diagnostic marker for hepatocellular carcinoma: A systematic review and meta-analysis. Pak. J. Med. Sci..

[87] Wang D., Sun X., Wei Y., Liang H., Yuan M., Jin F., Chen X., Liu Y., Zhang C.Y., Li L. (2018). Nuclear miR-122 directly regulates the biogenesis of cell survival oncomiR miR-21 at the posttranscriptional level. Nucleic Acids Res..

[88] Iwasaki A., Medzhitov R. (2004). Toll-like receptor control of the adaptive immune responses. Nat. Immunol..

[89] Wang J.P., Zhang Y., Wei X., Li J., Nan X.P., Yu H.T., Li Y., Wang P.Z., Bai X.F. (2010). Circulating Toll-like receptor (TLR) 2, TLR4, and regulatory T cells in patients with chronic hepatitis C. APMIS.

[90] Wei X.Q., Guo Y.W., Liu J.J., Wen Z.F., Yang S.J., Yao J.L. (2008). The significance of Toll-like receptor 4 (TLR4) expression in patients with chronic hepatitis B. Clin. Investig. Med..

[91] Machida K., Tsukamoto H., Mkrtchyan H., Duan L., Dynnyk A., Liu H.M., Asahina K., Govindarajan S., Ray R., Ou J.H. (2009). Toll-like receptor 4 mediates synergism between alcohol and HCV in hepatic oncogenesis involving stem cell marker Nanog. Proc. Natl. Acad. Sci. USA.

[92] Nishimura M., Naito S. (2005). Tissue-specific mRNA expression profiles of human toll-like receptors and related genes. Biol. Pharm. Bull..

[93] Yang J., Li M., Zheng Q.C. (2015). Emerging role of Toll-like receptor 4 in hepatocellular carcinoma. J. Hepatocell. Carcinoma.

[94] Shi L., Zheng X., Fan Y., Yang X., Li A., Qian J. (2019). The contribution of miR-122 to the innate immunity by regulating toll-like receptor 4 in hepatoma cells. BMC Gastroenterol..

[95] Wei X., Liu H., Li X., Liu X. (2019). Over-expression of MiR-122 promotes apoptosis of hepatocellular carcinoma via targeting TLR4. Ann. Hepatol..

[96] Tang N., Dou X., You X., Li Y., Li X., Liu G. (2021). Androgen Receptors Act as a Tumor Suppressor Gene to Suppress Hepatocellular Carcinoma Cells Progression via miR-122-5p/RABL6 Signaling. Front. Oncol..

[97] Petrizzo A., Caruso F.P., Tagliamonte M., Tornesello M.L., Ceccarelli M., Costa V., Aprile M., Esposito R., Ciliberto G., Buonaguro F.M. (2016). Identification and Validation of HCC-specific Gene Transcriptional Signature for Tumor Antigen Discovery. Sci. Rep..

[98] Sakamuro D., Eviner V., Elliott K.J., Showe L., White E., Prendergast G.C. (1995). c-Myc induces apoptosis in epithelial cells by both p53-dependent and p53-independent mechanisms. Oncogene.

[99] Prendergast G.C. (1999). Mechanisms of apoptosis by c-Myc. Oncogene.

[100] Dang C.V., O’Donnell K.A., Zeller K.I., Nguyen T., Osthus R.C., Li F. (2006). The c-Myc target gene network. Semin. Cancer Biol..

[101] Kawate S., Fukusato T., Ohwada S., Watanuki A., Morishita Y. (1999). Amplification of c-myc in hepatocellular carcinoma: Correlation with clinicopathologic features, proliferative activity and p53 overexpression. Oncology.

[102] Min Z., Xunlei Z., Haizhen C., Wenjing Z., Haiyan Y., Xiaoyun L., Jianyun Z., Xudong C., Aiguo S. (2021). The Clinicopathologic and Prognostic Significance of c-Myc Expression in Hepatocellular Carcinoma: A Meta-Analysis. Front. Bioinform..

[103] Jiang X., Liu Y., Wang G., Yao Y., Mei C., Wu X., Ma W., Yuan Y. (2020). Up-regulation of CLIC1 activates MYC signaling and forms a positive feedback regulatory loop with MYC in Hepatocellular carcinoma. Am. J. Cancer Res..

[104] Wang B., Hsu S.H., Wang X., Kutay H., Bid H.K., Yu J., Ganju R.K., Jacob S.T., Yuneva M., Ghoshal K. (2014). Reciprocal regulation of microRNA-122 and c-Myc in hepatocellular cancer: Role of E2F1 and transcription factor dimerization partner 2. Hepatology.

[105] Teng K.Y., Barajas J.M., Hu P., Jacob S.T., Ghoshal K. (2020). Role of B Cell Lymphoma 2 in the Regulation of Liver Fibrosis in miR-122 Knockout Mice. Biology.

[106] Alenzi F.Q., El-Nashar E.M., Al-Ghamdi S.S., Abbas M.Y., Hamad A.M., El-Saeed O.M., Wyse R.K., Lotfy M. (2010). Original Article: Investigation of Bcl-2 and PCNA in Hepatocellular Carcinoma: Relation to Chronic HCV. J. Egypt Natl. Canc. Inst..

[107] Fabregat I. (2009). Dysregulation of apoptosis in hepatocellular carcinoma cells. World J. Gastroenterol..

[108] Huang H., Zhu Y., Li S. (2015). MicroRNA-122 mimic transfection contributes to apoptosis in HepG2 cells. Mol. Med. Rep..

[109] Kojima K., Takata A., Vadnais C., Otsuka M., Yoshikawa T., Akanuma M., Kondo Y., Kang Y.J., Kishikawa T., Kato N. (2011). MicroRNA122 is a key regulator of alpha-fetoprotein expression and influences the aggressiveness of hepatocellular carcinoma. Nat. Commun..

[110] Wang S.C., Lin X.L., Li J., Zhang T.T., Wang H.Y., Shi J.W., Yang S., Zhao W.T., Xie R.Y., Wei F. (2014). MicroRNA-122 triggers mesenchymal-epithelial transition and suppresses hepatocellular carcinoma cell motility and invasion by targeting RhoA. PLoS ONE.

[111] Usman S., Waseem N.H., Nguyen T.K.N., Mohsin S., Jamal A., Teh M.T., Waseem A. (2021). Vimentin Is at the Heart of Epithelial Mesenchymal Transition (EMT) Mediated Metastasis. Cancers.

[112] Khalaf A.M., Fuentes D., Morshid A.I., Burke M.R., Kaseb A.O., Hassan M., Hazle J.D., Elsayes K.M. (2018). Role of Wnt/beta-catenin signaling in hepatocellular carcinoma, pathogenesis, and clinical significance. J. Hepatocell. Carcinoma.

[113] Luna J.M., Barajas J.M., Teng K.Y., Sun H.L., Moore M.J., Rice C.M., Darnell R.B., Ghoshal K. (2017). Argonaute CLIP Defines a Deregulated miR-122-Bound Transcriptome that Correlates with Patient Survival in Human Liver Cancer. Mol. Cell.

[114] Ahsani Z., Mohammadi-Yeganeh S., Kia V., Karimkhanloo H., Zarghami N., Paryan M. (2017). WNT1 Gene from WNT Signaling Pathway Is a Direct Target of miR-122 in Hepatocellular Carcinoma. Appl. Biochem. Biotechnol..

[115] Huge N., Sandbothe M., Schroder A.K., Stalke A., Eilers M., Schaffer V., Schlegelberger B., Illig T., Vajen B., Skawran B. (2020). Wnt status-dependent oncogenic role of BCL9 and BCL9L in hepatocellular carcinoma. Hepatol. Int..

[116] Hyeon J., Ahn S., Lee J.J., Song D.H., Park C.K. (2013). Prognostic Significance of BCL9 Expression in Hepatocellular Carcinoma. Korean J. Pathol..

[117] Dituri F., Mancarella S., Cigliano A., Chieti A., Giannelli G. (2019). TGF-beta as Multifaceted Orchestrator in HCC Progression: Signaling, EMT, Immune Microenvironment, and Novel Therapeutic Perspectives. Semin. Liver Dis..

[118] Fabregat I., Caballero-Diaz D. (2018). Transforming Growth Factor-beta-Induced Cell Plasticity in Liver Fibrosis and Hepatocarcinogenesis. Front. Oncol..

[119] Murillo M.M., Carmona-Cuenca I., Del Castillo G., Ortiz C., Roncero C., Sanchez A., Fernandez M., Fabregat I. (2007). Activation of NADPH oxidase by transforming growth factor-beta in hepatocytes mediates up-regulation of epidermal growth factor receptor ligands through a nuclear factor-kappaB-dependent mechanism. Biochem. J..

[120] Caja L., Sancho P., Bertran E., Fabregat I. (2011). Dissecting the effect of targeting the epidermal growth factor receptor on TGF-beta-induced-apoptosis in human hepatocellular carcinoma cells. J. Hepatol..

[121] Barry A.E., Baldeosingh R., Lamm R., Patel K., Zhang K., Dominguez D.A., Kirton K.J., Shah A.P., Dang H. (2020). Hepatic Stellate Cells and Hepatocarcinogenesis. Front. Cell Dev. Biol..

[122] Zeng C., Wang Y.L., Xie C., Sang Y., Li T.J., Zhang M., Wang R., Zhang Q., Zheng L., Zhuang S.M. (2015). Identification of a novel TGF-beta-miR-122-fibronectin 1/serum response factor signaling cascade and its implication in hepatic fibrogenesis. Oncotarget.

[123] Cheng B., Zhu Q., Lin W., Wang L. (2019). MicroRNA-122 inhibits epithelial-mesenchymal transition of hepatic stellate cells induced by the TGF-beta1/Smad signaling pathway. Exp. Ther. Med..

[124] Yin S., Fan Y., Zhang H., Zhao Z., Hao Y., Li J., Sun C., Yang J., Yang Z., Yang X. (2016). Differential TGFbeta pathway targeting by miR-122 in humans and mice affects liver cancer metastasis. Nat. Commun..

[125] Shyu Y.C., Lee T.L., Lu M.J., Chen J.R., Chien R.N., Chen H.Y., Lin J.F., Tsou A.P., Chen Y.H., Hsieh C.W. (2016). miR-122-mediated translational repression of PEG10 and its suppression in human hepatocellular carcinoma. J. Transl. Med..

[126] Xie T., Pan S., Zheng H., Luo Z., Tembo K.M., Jamal M., Yu Z., Yu Y., Xia J., Yin Q. (2018). PEG10 as an oncogene: Expression regulatory mechanisms and role in tumor progression. Cancer Cell Int..

[127] Tsou A.P., Chuang Y.C., Su J.Y., Yang C.W., Liao Y.L., Liu W.K., Chiu J.H., Chou C.K. (2003). Overexpression of a novel imprinted gene, PEG10, in human hepatocellular carcinoma and in regenerating mouse livers. J. Biomed. Sci..

[128] Ip W.K., Lai P.B., Wong N.L., Sy S.M., Beheshti B., Squire J.A., Wong N. (2007). Identification of PEG10 as a progression related biomarker for hepatocellular carcinoma. Cancer Lett..

[129] Bang H., Ha S.Y., Hwang S.H., Park C.K. (2015). Expression of PEG10 Is Associated with Poor Survival and Tumor Recurrence in Hepatocellular Carcinoma. Cancer Res. Treat..

[130] Zhang M., Sui C., Dai B., Shen W., Lu J., Yang J. (2017). PEG10 is imperative for TGF-beta1-induced epithelialmesenchymal transition in hepatocellular carcinoma. Oncol. Rep..

[131] Wang C., Xiao Y., Hu Z., Chen Y., Liu N., Hu G. (2008). PEG10 directly regulated by E2Fs might have a role in the development of hepatocellular carcinoma. FEBS Lett..

[132] Holstein E., Binder M., Mikulits W. (2018). Dynamics of Axl Receptor Shedding in Hepatocellular Carcinoma and Its Implication for Theranostics. Int. J. Mol. Sci..

[133] O’Bryan J.P., Frye R.A., Cogswell P.C., Neubauer A., Kitch B., Prokop C., Espinosa R., Le Beau M.M., Earp H.S., Liu E.T. (1991). axl, a transforming gene isolated from primary human myeloid leukemia cells, encodes a novel receptor tyrosine kinase. Mol. Cell Biol..

[134] Rankin E.B., Giaccia A.J. (2016). The Receptor Tyrosine Kinase AXL in Cancer Progression. Cancers.

[135] Zhu C., Wei Y., Wei X. (2019). AXL receptor tyrosine kinase as a promising anti-cancer approach: Functions, molecular mechanisms and clinical applications. Mol. Cancer.

[136] Hsu S.H., Wang B., Kutay H., Bid H., Shreve J., Zhang X., Costinean S., Bratasz A., Houghton P., Ghoshal K. (2013). Hepatic loss of miR-122 predisposes mice to hepatobiliary cyst and hepatocellular carcinoma upon diethylnitrosamine exposure. Am. J. Pathol..

[137] Chun K.H. (2022). Discovery of Cellular RhoA Functions by the Integrated Application of Gene Set Enrichment Analysis. Biomol. Ther..

[138] Grise F., Bidaud A., Moreau V. (2009). Rho GTPases in hepatocellular carcinoma. Biochim. Biophys. Acta.

[139] Bai Y., Xie F., Miao F., Long J., Huang S., Huang H., Lin J., Wang D., Yang X., Bian J. (2019). The diagnostic and prognostic role of RhoA in hepatocellular carcinoma. Aging.

[140] Tsubota A., Matsumoto K., Mogushi K., Nariai K., Namiki Y., Hoshina S., Hano H., Tanaka H., Saito H., Tada N. (2010). IQGAP1 and vimentin are key regulator genes in naturally occurring hepatotumorigenesis induced by oxidative stress. Carcinogenesis.

[141] Chen F., Zhu H.H., Zhou L.F., Wu S.S., Wang J., Chen Z. (2010). IQGAP1 is overexpressed in hepatocellular carcinoma and promotes cell proliferation by Akt activation. Exp. Mol. Med..

[142] Xia F.D., Wang Z.L., Chen H.X., Huang Y., Li J.D., Wang Z.M., Li X.Y. (2014). Differential expression of IQGAP1/2 in Hepatocellular carcinoma and its relationship with clinical outcomes. Asian Pac. J. Cancer Prev..

[143] Brill S., Li S., Lyman C.W., Church D.M., Wasmuth J.J., Weissbach L., Bernards A., Snijders A.J. (1996). The Ras GTPase-activating-protein-related human protein IQGAP2 harbors a potential actin binding domain and interacts with calmodulin and Rho family GTPases. Mol. Cell Biol..

[144] Noritake J., Watanabe T., Sato K., Wang S., Kaibuchi K. (2005). IQGAP1: A key regulator of adhesion and migration. J. Cell Sci..

[145] Bensenor L.B., Kan H.M., Wang N., Wallrabe H., Davidson L.A., Cai Y., Schafer D.A., Bloom G.S. (2007). IQGAP1 regulates cell motility by linking growth factor signaling to actin assembly. J. Cell Sci..

[146] Owen D., Campbell L.J., Littlefield K., Evetts K.A., Li Z., Sacks D.B., Lowe P.N., Mott H.R. (2008). The IQGAP1-Rac1 and IQGAP1-Cdc42 interactions: Interfaces differ between the complexes. J. Biol. Chem..

[147] Perez-Silva J.G., Espanol Y., Velasco G., Quesada V. (2016). The Degradome database: Expanding roles of mammalian proteases in life and disease. Nucleic Acids Res..

[148] Wetzel S., Seipold L., Saftig P. (2017). The metalloproteinase ADAM10: A useful therapeutic target?. Biochim. Biophys. Acta Mol. Cell Res..

[149] Dengler M., Staufer K., Huber H., Stauber R., Bantel H., Weiss K.H., Starlinger P., Pock H., Kloters-Plachky P., Gotthardt D.N. (2017). Soluble Axl is an accurate biomarker of cirrhosis and hepatocellular carcinoma development: Results from a large scale multicenter analysis. Oncotarget.

[150] Li Y., Brazzell J., Herrera A., Walcheck B. (2006). ADAM17 deficiency by mature neutrophils has differential effects on L-selectin shedding. Blood.

[151] Black R.A., Rauch C.T., Kozlosky C.J., Peschon J.J., Slack J.L., Wolfson M.F., Castner B.J., Stocking K.L., Reddy P., Srinivasan S. (1997). A metalloproteinase disintegrin that releases tumour-necrosis factor-alpha from cells. Nature.

[152] Sternlicht M.D., Sunnarborg S.W., Kouros-Mehr H., Yu Y., Lee D.C., Werb Z. (2005). Mammary ductal morphogenesis requires paracrine activation of stromal EGFR via ADAM17-dependent shedding of epithelial amphiregulin. Development.

[153] Castillo J., Erroba E., Perugorria M.J., Santamaria M., Lee D.C., Prieto J., Avila M.A., Berasain C. (2006). Amphiregulin contributes to the transformed phenotype of human hepatocellular carcinoma cells. Cancer Res..

[154] Ding X., Yang L.Y., Huang G.W., Wang W., Lu W.Q. (2004). ADAM17 mRNA expression and pathological features of hepatocellular carcinoma. World J. Gastroenterol..

[155] Mochizuki S., Okada Y. (2007). ADAMs in cancer cell proliferation and progression. Cancer Sci..

[156] Xiang Y., Liu L., Wang Y., Li B., Peng J., Feng D. (2020). ADAM17 promotes the invasion of hepatocellular carcinoma via upregulation MMP21. Cancer Cell Int..

[157] Yuan S., Lei S., Wu S. (2013). ADAM10 is overexpressed in human hepatocellular carcinoma and contributes to the proliferation, invasion and migration of HepG2 cells. Oncol. Rep..

[158] Zhang W., Liu S., Liu K., Wang Y., Ji B., Zhang X., Liu Y. (2014). A disintegrin and metalloprotease (ADAM)10 is highly expressed in hepatocellular carcinoma and is associated with tumour progression. J. Int. Med. Res..

[159] Csak T., Bala S., Lippai D., Satishchandran A., Catalano D., Kodys K., Szabo G. (2015). microRNA-122 regulates hypoxia-inducible factor-1 and vimentin in hepatocytes and correlates with fibrosis in diet-induced steatohepatitis. Liver Int..

[160] Hu L., Lau S.H., Tzang C.H., Wen J.M., Wang W., Xie D., Huang M., Wang Y., Wu M.C., Huang J.F. (2004). Association of Vimentin overexpression and hepatocellular carcinoma metastasis. Oncogene.

[161] Liu H., Li D., Zhou L., Kan S., He G., Zhou K., Wang L., Chen M., Shu W. (2020). LMNA functions as an oncogene in hepatocellular carcinoma by regulating the proliferation and migration ability. J. Cell Mol. Med..

[162] Abdelghany A.M., Rezk N.S., Osman M.M., Hamid A.I., Al-Breedy A.M., Abdelsattar H.A. (2018). Using Lamin B1 mRNA for the early diagnosis of hepatocellular carcinoma: A cross-sectional diagnostic accuracy study. F1000Res.

[163] Hu Z., Yang A., Su G., Zhao Y., Wang Y., Chai X., Tu P. (2016). Huaier restrains proliferative and invasive potential of human hepatoma SKHEP-1 cells partially through decreased Lamin B1 and elevated NOV. Sci. Rep..

[164] Nakatsuka T., Tateishi K., Kato H., Fujiwara H., Yamamoto K., Kudo Y., Nakagawa H., Tanaka Y., Ijichi H., Ikenoue T. (2021). Inhibition of histone methyltransferase G9a attenuates liver cancer initiation by sensitizing DNA-damaged hepatocytes to p53-induced apoptosis. Cell Death Dis..

[165] Qin J., Li Q., Zeng Z., Wu P., Jiang Y., Luo T., Ji X., Zhang Q., Hao Y., Chen L. (2018). Increased expression of G9A contributes to carcinogenesis and indicates poor prognosis in hepatocellular carcinoma. Oncol. Lett..

[166] Wei L., Chiu D.K., Tsang F.H., Law C.T., Cheng C.L., Au S.L., Lee J.M., Wong C.C., Ng I.O., Wong C.M. (2017). Histone methyltransferase G9a promotes liver cancer development by epigenetic silencing of tumor suppressor gene RARRES3. J. Hepatol..

[167] Yuan L.T., Lee W.J., Yang Y.C., Chen B.R., Yang C.Y., Chen M.W., Chen J.Q., Hsiao M., Chien M.H., Hua K.T. (2021). Histone Methyltransferase G9a-Promoted Progression of Hepatocellular Carcinoma Is Targeted by Liver-Specific Hsa-miR-122. Cancers.

[168] Fiorentino F., Mai A., Rotili D. (2021). Emerging Therapeutic Potential of SIRT6 Modulators. J. Med. Chem..

[169] Lee N., Ryu H.G., Kwon J.H., Kim D.K., Kim S.R., Wang H.J., Kim K.T., Choi K.Y. (2016). SIRT6 Depletion Suppresses Tumor Growth by Promoting Cellular Senescence Induced by DNA Damage in HCC. PLoS ONE.

[170] Ran L.K., Chen Y., Zhang Z.Z., Tao N.N., Ren J.H., Zhou L., Tang H., Chen X., Chen K., Li W.Y. (2016). SIRT6 Overexpression Potentiates Apoptosis Evasion in Hepatocellular Carcinoma via BCL2-Associated X Protein-Dependent Apoptotic Pathway. Clin. Cancer Res..

[171] Zhang C., Yu Y., Huang Q., Tang K. (2019). SIRT6 regulates the proliferation and apoptosis of hepatocellular carcinoma via the ERK1/2 signaling pathway. Mol. Med. Rep..

[172] Wang Y., Pan T., Wang H., Li L., Li J., Zhang D., Yang H. (2017). Overexpression of SIRT6 attenuates the tumorigenicity of hepatocellular carcinoma cells. Oncotarget.

[173] Zhang Z.G., Qin C.Y. (2014). Sirt6 suppresses hepatocellular carcinoma cell growth via inhibiting the extracellular signalregulated kinase signaling pathway. Mol. Med. Rep..

[174] Warburg O. (1956). On the origin of cancer cells. Science.

[175] Annibaldi A., Widmann C. (2010). Glucose metabolism in cancer cells. Curr. Opin. Clin. Nutr. Metab. Care.

[176] Tenen D.G., Chai L., Tan J.L. (2021). Metabolic alterations and vulnerabilities in hepatocellular carcinoma. Gastroenterol. Rep..

[177] Bidkhori G., Benfeitas R., Klevstig M., Zhang C., Nielsen J., Uhlen M., Boren J., Mardinoglu A. (2018). Metabolic network-based stratification of hepatocellular carcinoma reveals three distinct tumor subtypes. Proc. Natl. Acad. Sci. USA.

[178] Bjornson E., Mukhopadhyay B., Asplund A., Pristovsek N., Cinar R., Romeo S., Uhlen M., Kunos G., Nielsen J., Mardinoglu A. (2015). Stratification of Hepatocellular Carcinoma Patients Based on Acetate Utilization. Cell Rep..

[179] Nwosu Z.C., Battello N., Rothley M., Pioronska W., Sitek B., Ebert M.P., Hofmann U., Sleeman J., Wolfl S., Meyer C. (2018). Liver cancer cell lines distinctly mimic the metabolic gene expression pattern of the corresponding human tumours. J. Exp. Clin. Cancer Res..

[180] Liu A.M., Xu Z., Shek F.H., Wong K.F., Lee N.P., Poon R.T., Chen J., Luk J.M. (2014). miR-122 targets pyruvate kinase M2 and affects metabolism of hepatocellular carcinoma. PLoS ONE.

[181] Gatfield D., Le Martelot G., Vejnar C.E., Gerlach D., Schaad O., Fleury-Olela F., Ruskeepaa A.L., Oresic M., Esau C.C., Zdobnov E.M. (2009). Integration of microRNA miR-122 in hepatic circadian gene expression. Genes Dev..

[182] Elmen J., Lindow M., Silahtaroglu A., Bak M., Christensen M., Lind-Thomsen A., Hedtjarn M., Hansen J.B., Hansen H.F., Straarup E.M. (2008). Antagonism of microRNA-122 in mice by systemically administered LNA-antimiR leads to up-regulation of a large set of predicted target mRNAs in the liver. Nucleic Acids Res..

[183] Niu Y., Lin Z., Wan A., Sun L., Yan S., Liang H., Zhan S., Chen D., Bu X., Liu P. (2021). Loss-of-Function Genetic Screening Identifies Aldolase A as an Essential Driver for Liver Cancer Cell Growth Under Hypoxia. Hepatology.

[184] Noguchi T., Inoue H., Tanaka T. (1986). The M1- and M2-type isozymes of rat pyruvate kinase are produced from the same gene by alternative RNA splicing. J. Biol. Chem..

[185] Noguchi T., Yamada K., Inoue H., Matsuda T., Tanaka T. (1987). The L- and R-type isozymes of rat pyruvate kinase are produced from a single gene by use of different promoters. J. Biol. Chem..

[186] Zahra K., Dey T., Ashish, Mishra S.P., Pandey U. (2020). Pyruvate Kinase M2 and Cancer: The Role of PKM2 in Promoting Tumorigenesis. Front. Oncol..

[187] Li T.E., Wang S., Shen X.T., Zhang Z., Chen M., Wang H., Zhu Y., Xu D., Hu B.Y., Wei R. (2020). PKM2 Drives Hepatocellular Carcinoma Progression by Inducing Immunosuppressive Microenvironment. Front. Immunol..

[188] Wong C.C., Au S.L., Tse A.P., Xu I.M., Lai R.K., Chiu D.K., Wei L.L., Fan D.N., Tsang F.H., Lo R.C. (2014). Switching of pyruvate kinase isoform L to M2 promotes metabolic reprogramming in hepatocarcinogenesis. PLoS ONE.

[189] El-Ashmawy N.E., El-Bahrawy H.A., Shamloula M.M., El-Feky O.A. (2014). Biochemical/metabolic changes associated with hepatocellular carcinoma development in mice. Tumour Biol..

[190] Banka S., Newman W.G. (2013). A clinical and molecular review of ubiquitous glucose-6-phosphatase deficiency caused by G6PC3 mutations. Orphanet J. Rare Dis..

[191] Barajas J.M., Reyes R., Guerrero M.J., Jacob S.T., Motiwala T., Ghoshal K. (2018). The role of miR-122 in the dysregulation of glucose-6-phosphate dehydrogenase (G6PD) expression in hepatocellular cancer. Sci. Rep..

[192] Xu H., He J.H., Xiao Z.D., Zhang Q.Q., Chen Y.Q., Zhou H., Qu L.H. (2010). Liver-enriched transcription factors regulate microRNA-122 that targets CUTL1 during liver development. Hepatology.

[193] Marrone A.K., Tryndyak V., Beland F.A., Pogribny I.P. (2016). MicroRNA Responses to the Genotoxic Carcinogens Aflatoxin B1 and Benzo[a]pyrene in Human HepaRG Cells. Toxicol. Sci..

[194] Deng X.G., Qiu R.L., Wu Y.H., Li Z.X., Xie P., Zhang J., Zhou J.J., Zeng L.X., Tang J., Maharjan A. (2014). Overexpression of miR-122 promotes the hepatic differentiation and maturation of mouse ESCs through a miR-122/FoxA1/HNF4a-positive feedback loop. Liver Int..

[195] Li M., Tang Y., Wu L., Mo F., Wang X., Li H., Qi R., Zhang H., Srivastava A., Ling C. (2017). The hepatocyte-specific HNF4alpha/miR-122 pathway contributes to iron overload-mediated hepatic inflammation. Blood.

[196] Li Z.Y., Xi Y., Zhu W.N., Zeng C., Zhang Z.Q., Guo Z.C., Hao D.L., Liu G., Feng L., Chen H.Z. (2011). Positive regulation of hepatic miR-122 expression by HNF4alpha. J. Hepatol..

[197] Lu H., Lei X., Liu J., Klaassen C. (2017). Regulation of hepatic microRNA expression by hepatocyte nuclear factor 4 alpha. World J. Hepatol..

[198] Yang Y.M., Lee C.G., Koo J.H., Kim T.H., Lee J.M., An J., Kim K.M., Kim S.G. (2015). Galpha12 overexpressed in hepatocellular carcinoma reduces microRNA-122 expression via HNF4alpha inactivation, which causes c-Met induction. Oncotarget.

[199] Aydin Y., Kurt R., Song K., Lin D., Osman H., Youngquist B., Scott J.W., Shores N.J., Thevenot P., Cohen A. (2019). Hepatic Stress Response in HCV Infection Promotes STAT3-Mediated Inhibition of HNF4A-miR-122 Feedback Loop in Liver Fibrosis and Cancer Progression. Cancers.

[200] Xu L., Hui L., Wang S., Gong J., Jin Y., Wang Y., Ji Y., Wu X., Han Z., Hu G. (2001). Expression profiling suggested a regulatory role of liver-enriched transcription factors in human hepatocellular carcinoma. Cancer Res..

[201] Zeng C., Wang R., Li D., Lin X.J., Wei Q.K., Yuan Y., Wang Q., Chen W., Zhuang S.M. (2010). A novel GSK-3 beta-C/EBP alpha-miR-122-insulin-like growth factor 1 receptor regulatory circuitry in human hepatocellular carcinoma. Hepatology.

[202] Zeng C., Sang Y., Wang F.Y., Zhuang S.M. (2020). Opposing roles of C/EBPalpha and eEF1A1 in Sp1-regulated miR-122 transcription. RNA Biol..

[203] Song K., Han C., Zhang J., Lu D., Dash S., Feitelson M., Lim K., Wu T. (2013). Epigenetic regulation of MicroRNA-122 by peroxisome proliferator activated receptor-gamma and hepatitis b virus X protein in hepatocellular carcinoma cells. Hepatology.

[204] Huang X.F., Zhao W.Y., Huang W.D. (2015). FXR and liver carcinogenesis. Acta Pharmacol. Sin..

[205] He J., Zhao K., Zheng L., Xu Z., Gong W., Chen S., Shen X., Huang G., Gao M., Zeng Y. (2015). Upregulation of microRNA-122 by farnesoid X receptor suppresses the growth of hepatocellular carcinoma cells. Mol. Cancer.

[206] Huang Z., Zhou J.K., Peng Y., He W., Huang C. (2020). The role of long noncoding RNAs in hepatocellular carcinoma. Mol. Cancer.

[207] Ma J., Li T., Han X., Yuan H. (2018). Knockdown of LncRNA ANRIL suppresses cell proliferation, metastasis, and invasion via regulating miR-122-5p expression in hepatocellular carcinoma. J. Cancer Res. Clin. Oncol..

[208] Wan Y., Li M., Huang P. (2019). LINC01296 promotes proliferation, migration, and invasion of HCC cells by targeting miR-122-5P and modulating EMT activity. Onco. Targets Ther..

[209] Zhang L., Wang Y., Sun J., Ma H., Guo C. (2019). LINC00205 promotes proliferation, migration and invasion of HCC cells by targeting miR-122-5p. Pathol. Res. Pract..

[210] Yang X., Sun L., Wang L., Yao B., Mo H., Yang W. (2019). LncRNA SNHG7 accelerates the proliferation, migration and invasion of hepatocellular carcinoma cells via regulating miR-122-5p and RPL4. Biomed. Pharmacother..

[211] Liang Y., Zhang D., Zheng T., Yang G., Wang J., Meng F., Liu Y., Zhang G., Zhang L., Han J. (2020). lncRNA-SOX2OT promotes hepatocellular carcinoma invasion and metastasis through miR-122-5p-mediated activation of PKM2. Oncogenesis.

[212] Yang X., Yao B., Niu Y., Chen T., Mo H., Wang L., Guo C., Yao D. (2019). Hypoxia-induced lncRNA EIF3J-AS1 accelerates hepatocellular carcinoma progression via targeting miR-122-5p/CTNND2 axis. Biochem. Biophys. Res. Commun..

[213] Wu X., Yang Y., Huang Y., Chen Y., Wang T., Wu S., Tong L., Wang Y., Lin L., Hao M. (2018). RNA-binding protein AUF1 suppresses miR-122 biogenesis by down-regulating Dicer1 in hepatocellular carcinoma. Oncotarget.

